# Glycans function as a Golgi export signal to promote the constitutive exocytic trafficking

**DOI:** 10.1074/jbc.RA120.014476

**Published:** 2020-08-21

**Authors:** Xiuping Sun, Hieng Chiong Tie, Bing Chen, Lei Lu

**Affiliations:** School of Biological Sciences, Nanyang Technological University, Singapore

**Keywords:** Golgi, Golgi export, O-glycosylation, N-glycosylation, secretory pathway, protein trafficking (Golgi), membrane trafficking, intracellular trafficking, membrane protein, glycosylation, exocytosis, imaging, constitutive exocytosis, Golgi localization

## Abstract

Most proteins in the secretory pathway are glycosylated. However, the role of glycans in membrane trafficking is still unclear. Here, we discovered that transmembrane secretory cargos, such as interleukin 2 receptor α subunit or Tac, transferrin receptor, and cluster of differentiation 8a, unexpectedly displayed substantial Golgi localization when their *O*-glycosylation was compromised. By quantitatively measuring their Golgi residence times, we found that the observed Golgi localization of *O*-glycan–deficient cargos is due to their slow Golgi export. Using a superresolution microscopy method that we previously developed, we revealed that *O*-glycan–deficient Tac chimeras localize at the interior of the *trans*-Golgi cisternae. *O*-Glycans were observed to be both necessary and sufficient for the efficient Golgi export of Tac chimeras. By sequentially introducing *O*-glycosylation sites to ST6GAL1, we demonstrated that *O*-glycan's effect on Golgi export is probably additive. Finally, the finding that *N*-glycosylated GFP substantially reduces the Golgi residence time of a Tac chimera suggests that *N*-glycans might have a similar effect. Therefore, both *O*- and *N*-glycans might function as a generic Golgi export signal at the *trans*-Golgi to promote the constitutive exocytic trafficking.

In the secretory pathway, newly synthesized membrane proteins (cargos) are exported from the endoplasmic reticulum (ER) toward the Golgi, where they sequentially pass through cisternae of the Golgi stack. Once reaching the *trans*-side of the Golgi, cargos are packed into membrane carries destined for the plasma membrane (PM) or endolysosomes (1–3). It is well-accepted that protein trafficking is mediated by a signal, usually a cytosolic short stretch of amino acids (AAs) that is recognized by diverse trafficking machineries. In the secretory pathway, the efficient ER export of many cargos can be facilitated by COPII coat–binding ER export signals (4, 5). At the *trans*-side of the Golgi, secretory cargos that possess clathrin adaptor–interacting signals are targeted to the endolysosome in clathrin-coated vesicles (1–3, 6). Without such targeting or export signals, the majority of secretory cargos, such as vesicular stomatitis glycoprotein G (VSVG), follow the by default constitutive exocytic pathway and are exported from the Golgi in noncoated membrane carriers (7–9). So far, the generic Golgi export signal has not been identified for the constitutive pathway, although a putative signal was reported in a viral membrane protein (10). Lacking a conventional coat-like machinery, it is also unclear how these cargos are sorted from Golgi transmembrane-resident proteins (hereafter referred to as residents) and recruited to the exocytic membrane carriers.

Almost all secretory proteins are glycosylated in the ER and/or Golgi. However, the roles of polysaccharide chains or glycans in the protein secretion are still unclear. The best-known example is the mannose 6-phosphate (M6P)-modified glycan, which specifically attaches to the soluble hydrolytic enzymes of the lysosome (11, 12). At the TGN, M6P is recognized by M6P receptor, which subsequently concentrates lysosomal enzymes to clathrin-coated vesicles targeting to the endolysosome. As another example, at the ER exit site, the cargo receptor, ERGIC53, binds to the high-mannose *N*-glycans of several soluble secretory proteins, such as coagulation factor V and VII, for their efficient ER export through COPII-coated vesicles (13, 14).

Although the molecular mechanism behind is still obscure, available data indicate that glycans are also involved in the sorting and trafficking of secretory cargos to the PM. In cargos without basolateral PM sorting signals, glycans have been demonstrated by multiple research groups to act as an apical PM-targeting signal in polarized epithelial cells (15, 16). Notably, an attempt has been made by Gut *et al.* (17) to determine the role of *N*-glycosylation in the Golgi-to-PM trafficking. By employing reporters with mutated basolateral PM-targeting signals, they found that artificially introducing *N*-glycans targeted Golgi-arrested occludin and ERGIC53 chimeras to the PM, whereas abolishing the native *N*-glycosylation of Fc receptor by tunicamycin arrested its exocytic trafficking in the Golgi. In the budding yeast, Proszynski *et al.* (18) found that compromising the *O*-glycosylation of a PM transmembrane protein, Fus1p, caused its accumulation in the late Golgi. Furthermore, they noted that the introduction of an *O*-glycosylation site to the Golgi-localized Fus1p mutant rescued its transport to the PM. Despite these works, according to our knowledge, the role of *O*- and *N*-glycans on the constitutive secretory transport of Golgi is still unclear and has not been systematically studied. Here, we first found that cargos lacking *O*-glycosylation can accumulate in the Golgi due to their slow Golgi export. By quantitatively measuring the Golgi residence times of various transmembrane secretory reporters, we subsequently uncovered that both *O*- and *N*-glycans can function as a generic Golgi export signal at the *trans*-Golgi for constitutive exocytic trafficking.

## Results

### Tac lacking the luminal region (Tac-TC) accumulates in the Golgi

**Figure 1.**
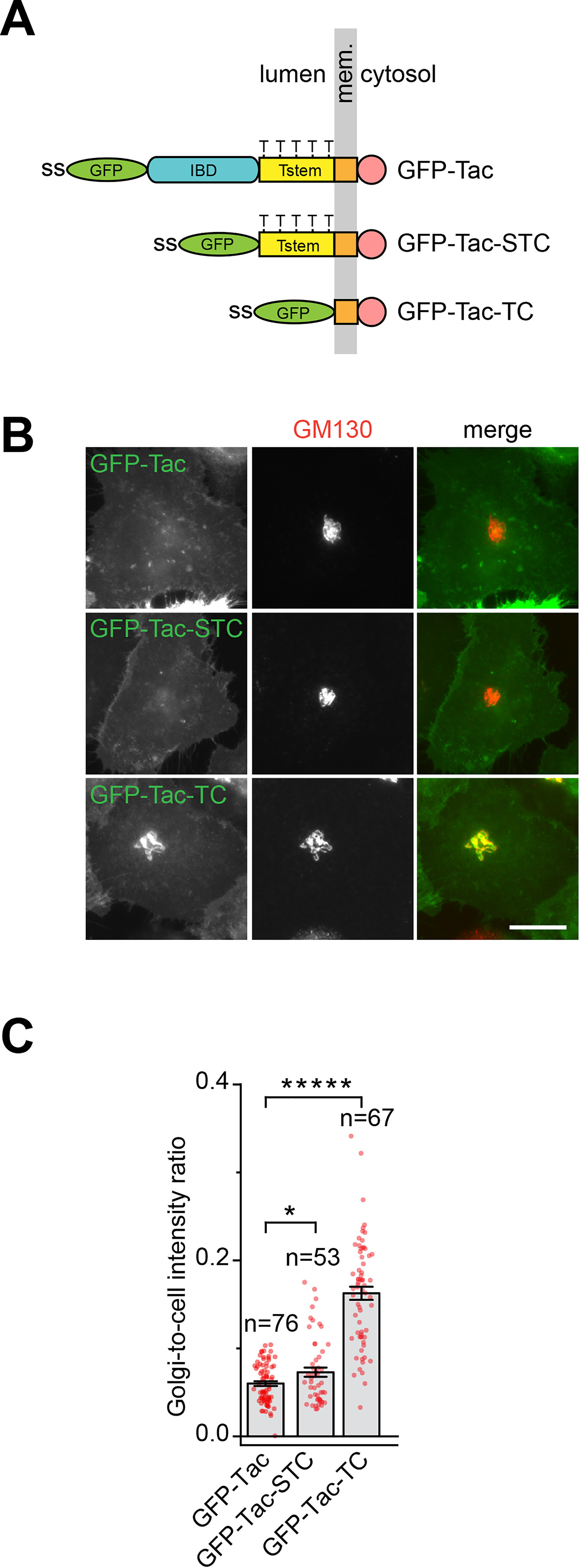
**Tac-TC localizes to the Golgi.** *A*, schematic diagram showing the organization of Tac truncation clones. *Mem*., membrane; *ss*, signal sequence. Five Thr residues (*T*) that are potentially under mucin-type *O*-glycosylation are indicated in Tstem. *B*, localization of Tac truncation clones. HeLa cells transiently expressing the indicated Tac truncation clone were immunostained for endogenous GM130 (a Golgi marker). *Scale bar*, 20 μm. *C*, Golgi-to-cell intensity ratios of Tac truncation clones. The images from *B* were quantified. *Error bars*, S.E.; *p* values are from a *t* test (unpaired and two-tailed); *****, *p* ≤ 0.000005; *, *p* ≤ 0.05; *n*, number of quantified cells.

During our study of secretory cargos, we focused on the cell surface receptor, interleukin 2 (IL2) receptor α subunit, or Tac. The expression of Tac is restricted to immune cells. Devoid of known sorting signals, it is commonly used as a type I transmembrane reporter for the study of the membrane trafficking. The luminal region of Tac comprises the IL2-binding domain (IBD) and stem region (Fig. 1*A*). The stem region is a short stretch of ∼30 AAs connecting the transmembrane domain and the extracellular IBD. GFP-tagged truncation clones were constructed to explore roles of different regions in Tac's secretory trafficking (Fig. 1*A*). As expected, full-length Tac was mainly found on the PM at the steady state, with no colocalization with the Golgi marker GM130 (Fig. 1*B*). However, in addition to the PM, we were surprised to find a predominant Golgi localization of Tac-TC (Fig. 1*B*), a truncation mutant missing the entire luminal region. Tac-STC, the IBD deletion clone, displayed an intermediate phenotype, in which roughly half of expressing cells had the same localization pattern as Tac (Fig. 1*B*), whereas the remaining showed the Golgi localization (Fig. S1*A*). Consistent with the visual assessment, the Golgi-to-cell intensity ratio of Tac-STC is between that of Tac and Tac-TC. Further investigation of Tac-STC on its Golgi localization is described below. Similar to a conventional Golgi resident, such as B4GALT1 (hereafter GalT), when the Golgi was disassembled by brefeldin A (19), the Golgi pool of Tac-TC redistributed to the ER (Fig. S1, *B* and *C*); when the Golgi was “dispersed” by the microtubule inhibitor, nocodazole (20), Tac-TC also followed GalT to the Golgi mini-stack at the ER exit site (Fig. S1*D*). The normal Golgi localization of Tac-TC was restored when brefeldin A or nocodazole was washed out (Fig. S1, *B–D*). The protein synthesis inhibitor, cycloheximide (CHX), was added throughout the treatment and washout to minimize the interference of the newly synthesized Tac-TC. In summary, our observations demonstrate that Tac-TC of the Golgi pool behaves like a conventional Golgi resident.

### The Golgi export of Tac-TC is compromised

**Figure 2.**
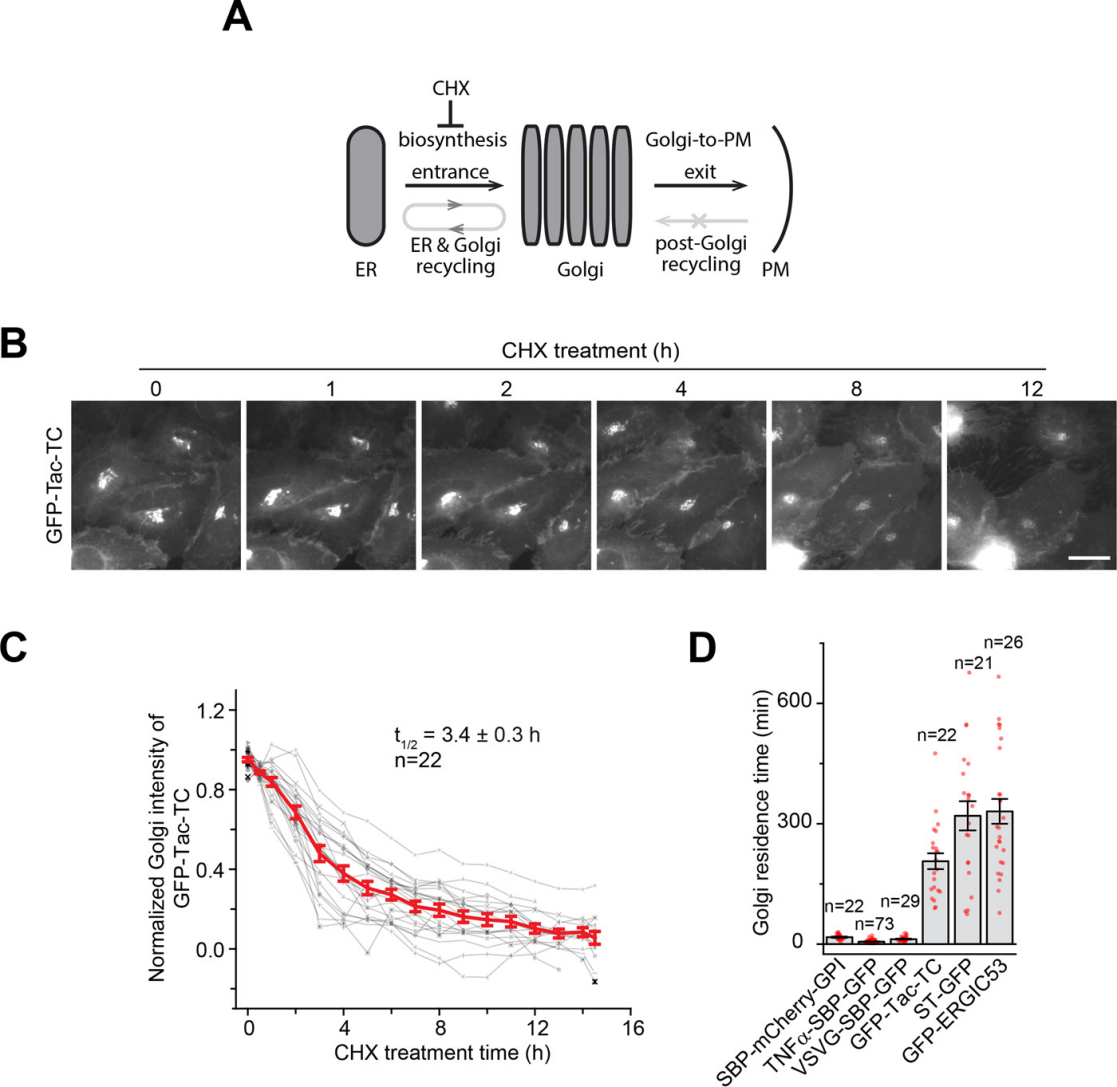
**The Golgi export of Tac-TC is compromised.** *A*, schematic diagram showing various trafficking pathways that contribute to a cargo's Golgi pool. See “Results” for details. *B–D*, the Golgi residence time of Tac-TC is significantly longer than that of conventional secretory cargos. In *B*, HeLa cells transiently co-expressing GFP-Tac-TC and GalT-mCherry were treated with CHX and imaged live. Only GFP-Tac-TC images are shown. *Scale bar*, 20 μm. In *C*, the total fluorescence intensity within the Golgi was quantified at each time point and subsequently plotted. Each intensity series was normalized and fitted to the first-order exponential decay function to acquire the Golgi residence time, *t*_½_. *Gray*, individual time series; *red*, averaged time series. *D*, Golgi residence times of secretory cargos and Golgi residents. See Fig. S2 (*D–M*). *Error bars*, S.E.; *n*, number of quantified cells.

Tac has not been known as a Golgi resident. A Golgi resident is usually expected to have a retrieval and/or retention mechanism to maintain its steady-state localization at the Golgi (21, 22). What is the mechanism behind the Golgi localization of Tac-TC? A secretory cargo's Golgi localization is determined by its entrance and exit (or export) of the Golgi (Fig. 2*A*). For a conventional secretory cargo such as Tac, the Golgi entrance at the steady state is primarily contributed by the ER-to-Golgi transport of newly synthesized protein, whereas the exit is contributed by the Golgi-to-PM trafficking. Although recycling can possibly occur between the Golgi and ER (*e.g.* the retrograde followed by the anterograde or ER-to-Golgi trafficking), the recycled fraction is likely to be small. Most importantly, if a cargo resides in the Golgi at the steady state for a sufficiently long time, the cycling between the Golgi and ER reaches a semi-steady state and hence does not produce a substantial net change in the cargo's Golgi pool.

We also studied the possibility of the post-Golgi retrieval of our reporters to the Golgi. It is known that VSVG does not return to the Golgi from the PM (23). Using the internalization of the surface-bound antibody, constitutive secretory cargos such as Tac, cluster of differentiation 8a (CD8a), transferrin receptor (TfR), tumor necrosis factor α (TNFα), and glycosylphosphatidylinositol-anchored mCherry (mCherry-GPI), did not return to the Golgi, once reaching the PM (Fig. S2*A*), consistent with their lack of Golgi-targeting signals. Interestingly, we found that post-Golgi-localized ST6GAL1 (hereafter ST) and ERGIC53 did not target to the Golgi from the PM and the endolysosome either (the significance of which will be discussed elsewhere) (Fig. S2, *B* and *C*). Hence, the pre- and post-Golgi recycling or retrieval are not considered in the Golgi localization of cargos used in this study.

At the steady state, we can assume that the cargo's Golgi entrance velocity is a constant, which is determined by its rate of biosynthesis, and its Golgi export velocity follows the first-order kinetics. When the velocity of the export is equal to that of the entrance, the Golgi pool reaches dynamic equilibrium, and its size is expected to be in a reverse relationship with the Golgi export rate constant. Hence, a cargo that slowly exits the Golgi can have a significant Golgi localization, and, once the protein synthesis is blocked, the cargo's Golgi pool gradually decreases by following the exponential decay function (7, 24).

To test the hypothesis that the Golgi localization of Tac-TC is due to its slow Golgi export, we measured the *t*_½_ of Tac-TC's Golgi export and compared it with those of Golgi residents and conventional secretory cargos. To that end, we live-imaged cells expressing GFP-tagged proteins under the treatment of CHX (Fig. 2*B*). The *t*_½_, referred to as the Golgi residence time hereafter, was subsequently acquired by fitting the Golgi intensity decay data using the first-order exponential function (Fig. 2*C*). The usage of the Golgi residence time as a Golgi export metric is advantageous, as it should be independent of the cargo's expression level and post-Golgi fates, such as degradation and cleavage. The Golgi residence times of ST and ERGIC53 were similarly measured (Fig. S2, *D* and *E*). RUSH (retention using selective hooks) reporters (25) were employed to measure the Golgi residence times of constitutive secretory cargos, including mCherry-GPI, TNFα, and VSVG. They were first accumulated in the Golgi by 20 °C temperature block (26), and the resulting Golgi pools were subsequently live-imaged at 37 °C (Fig. S2, *F–H*), at which RUSH reporters started synchronously leaving the Golgi for the PM. Altogether, our quantitative data demonstrate that, although significantly less than those of ST (5.3 h) and ERGIC53 (5.5 h) (Fig. S2, *I* and *J*), the Golgi residence time of Tac-TC (3.4 h) is ≥10-fold those of mCherry-GPI, TNFα, and VSVG (Fig. 2*D* and Fig. S2 (*K–M*)). Therefore, Tac-TC differs from conventional secretory cargos in its slow Golgi export, which likely results in its apparent Golgi localization.

### The O-glycan is essential for the efficient Golgi export of Tac

From the Golgi localization results of Tac truncation clones (Fig. 1*B*), we reasoned that the stem region should be essential for the efficient Golgi export. The most significant feature within this region is the presence of multiple Thr residues, which undergo mucin-type *O*-glycosylation at the Golgi (27). We hence asked if the *O*-glycan can serve as a Golgi export signal for Tac.

**Figure 3.**
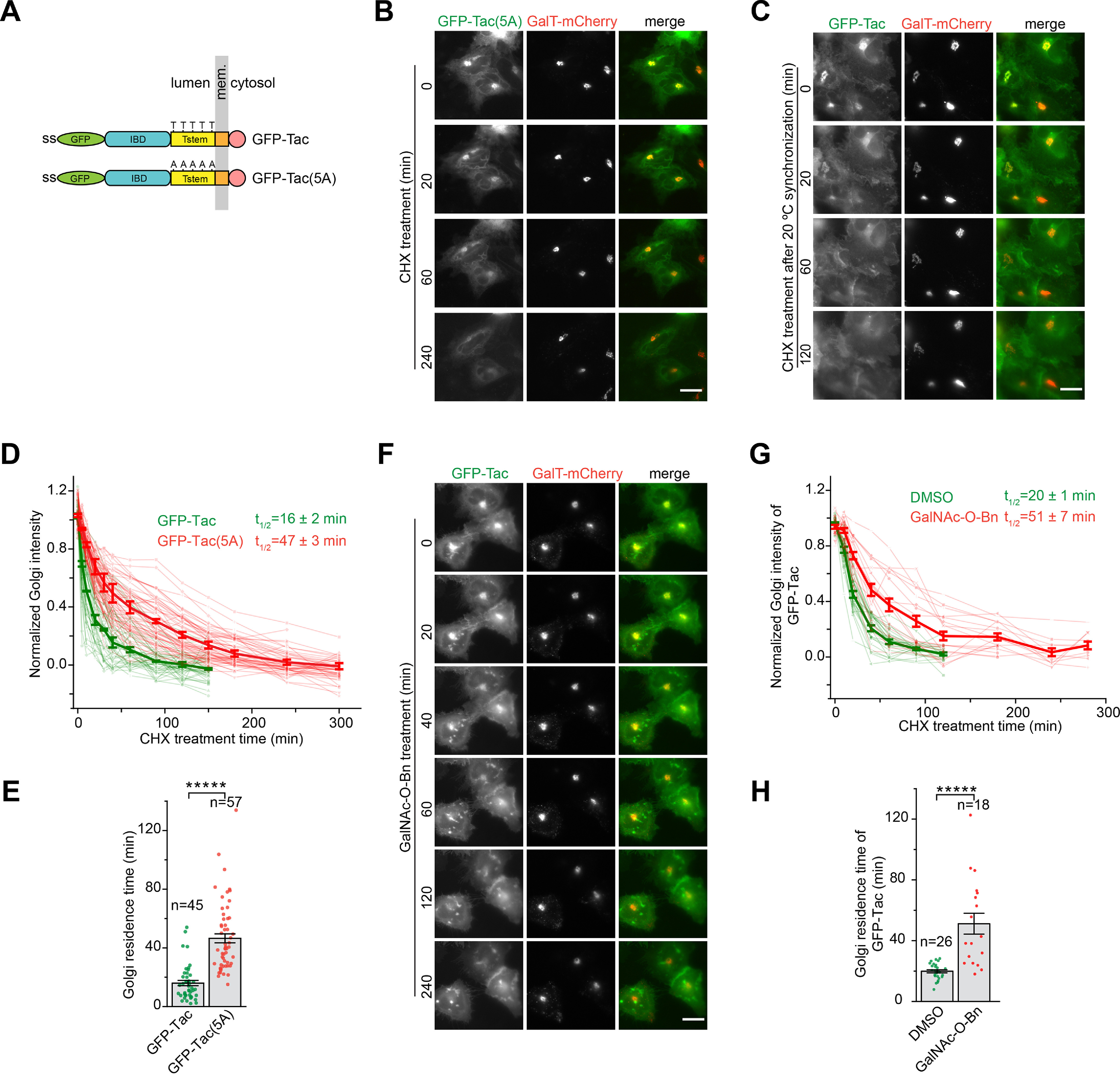
**The *O*-glycan at the stem region is essential for the efficient Golgi export of Tac; HeLa cells were used.** *A*, schematic diagram showing the domain organization and glycosylation or mutation sites of GFP-Tac and GFP-Tac(5A). The *panel i*s organized as described for Fig. 1*A*. Five Thr residues (*T*) and their corresponding Ala mutations (*A*) are indicated in Tstem of GFP-Tac and GFP-Tac(5A), respectively. *B–E*, the Golgi residence time of Tac(5A) is significantly longer than that of Tac. In *B*, cells transiently co-expressing GFP-Tac(5A) and GalT-mCherry were imaged live in the presence of CHX. In *C*, cells transiently co-expressing GFP-Tac and GalT-mCherry were incubated at 20 °C for 4 h and subsequently warmed up to 37 °C and imaged live in the presence of CHX (see “Experimental procedures”). The images acquired in *B* and *C* were quantified and plotted in *D* and *E*, similar to Fig. 2 (*C* and *D*). *F–H*, inhibiting the *O*-glycosylation significantly prolongs the Golgi residence time of Tac. In *F*, cells transiently co-expressing GFP-Tac and GalT-mCherry were first incubated with GalNAc-*O*-Bn for 20 h and subsequently imaged live in the presence of CHX. The images acquired in *F* were quantified and plotted in *G* and *H*, similar to Fig. 2 (*C* and *D*). *Scale bar*, 20 μm; *error bars*, mean ± S.E.; *p* values are from a *t* test (unpaired and two-tailed); *****, *p* ≤ 0.000005; *n*, number of quantified cells.

To test this hypothesis, we inhibited the *O*-glycosylation of Tac using two approaches. First, an *O*-glycosylation–negative Tac mutant, Tac(5A), was generated by mutating all 5 Thr residues within the stem region (Fig. 3*A*), which were previously known to be *O*-glycosylated (27). When expressed in cells, Tac(5A) displayed a strong Golgi localization (Fig. 3*B*, 0 min), in contrast to Tac (Fig. 1*B*). To measure the Golgi residence time of Tac, which does not display a steady-state Golgi localization, the 20 °C synchronization protocol was used to first accumulate GFP-Tac in the Golgi before live-imaging at 37 °C in the presence of CHX (Fig. 3*C*). Our data showed that the Golgi residence time of Tac(5A) (47 min) is almost 3-fold that of Tac (16 min) (Fig. 3, *D* and *E*). To rule out the effect of the pH variation across different Golgi regions on the GFP fluorescence (28) and the potential structural hindrance of the luminal region by the luminally tagged GFP, we prepared C-terminally or cytosolically tagged Tac and Tac(5A) (Fig. S3*A*). We found that the Golgi residence times of C-terminally tagged Tac and Tac(5A) were very similar to the corresponding N-terminally tagged ones (Fig. S3, *B* and *C*), therefore suggesting that the pH and hindrance effect of GFP might be insignificant. Second, we took advantage of a small-molecule inhibitor, benzyl 2-acetamido-2-deoxy-α-d-galactopyranoside (GalNAc-*O*-Bn), which compromises the mucin-type *O*-glycosylation by blocking the extension of *O*-GalNAc, the first sugar that is covalently linked to the protein (29). We confirmed the inhibitory effect of GalNAc-*O*-Bn on the *O*-glycosylation of Tac by its gel migration profile (Fig. S3*D*). When cells expressing GFP-Tac were treated with GalNAc-*O*-Bn, Tac exhibited a substantial Golgi localization, and its Golgi residence time (51 min) is 2.6-fold that of DMSO control (20 min) (Fig. 3, *F–H*), which was acquired by the 20 °C synchronization protocol described above. Thus, extended *O*-glycans (hereafter *O*-glycans) can substantially promote the Golgi export of Tac compared with *O*-GalNAc, which results from prolonged GalNAc-*O*-Bn treatment. Consistent with our result, it was previously reported that *O*-glycosylation–deficient Tac accumulated intracellularly and had little cell surface expression (30); in the budding yeast, Fus1p, a cell surface type I transmembrane protein, was found to require the *O*-glycosylation at its juxtamembrane region for its post-Golgi trafficking to the PM (18). We further investigated why the stem region containing Tac-STC displayed a mixed phenotype with a substantial fraction of cells showing the Golgi localization (Fig. 1*B*). The gel migration profile of Tac-STC and its 5A mutant revealed that only a small fraction of Tac-STC, 10% by quantifying band intensities in *lane 1* and *3*, is *O*-glycosylated (Fig. S3*E*). Hence, its Golgi localization is probably due to the lack of *O*-glycosylation in Tac-STC. Together, our observations suggest that the *O*-glycan at the stem region might be necessary for the efficient Golgi export of Tac.

### The O-glycan might be essential for the efficient Golgi export of generic cargos

**Figure 4.**
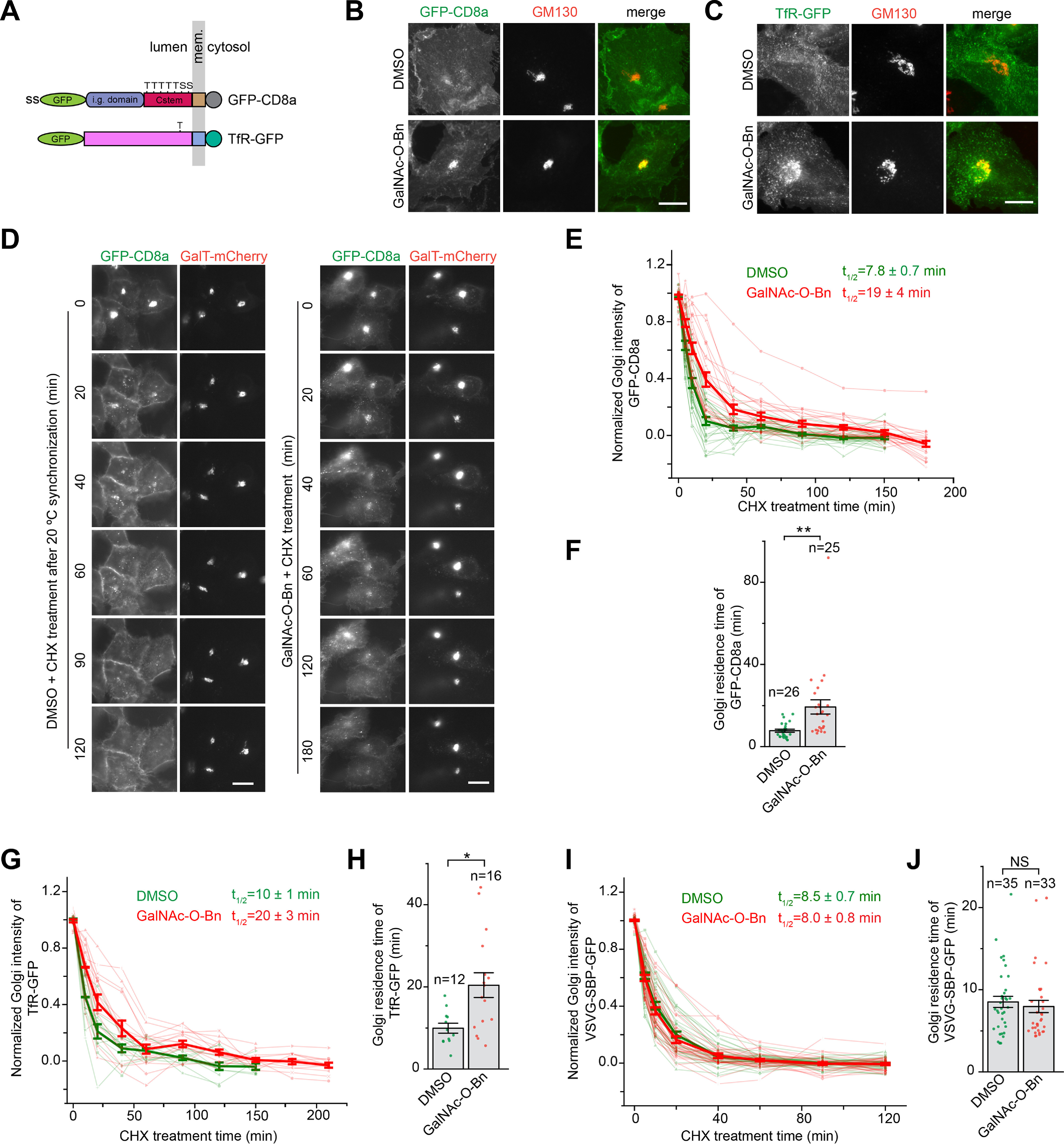
**The *O*-glycan is required for the efficient Golgi export of CD8a and TfR; HeLa cells were used.** *A*, schematic diagram showing the domain organization and potential *O*-glycosylation sites of CD8a and TfR. The *panel i*s organized as described for Fig. 1*A*. *i.g.*, immunoglobulin. Thr (*T*) or Ser (*S*) residues that are potentially under mucin-type *O*-glycosylation are indicated. *B* and *C*, a substantial amount of CD8a and TfR is accumulated in the Golgi when their *O*-glycosylation is inhibited. Cells transiently expressing GFP-CD8a or TfR-GFP were treated with DMSO or GalNAc-*O*-Bn for 20 h before immunostaining of endogenous GM130. *D–J*, inhibiting the *O*-glycosylation significantly prolongs the Golgi residence times of CD8a and TfR but not VSVG. In *D*, cells transiently co-expressing GFP-CD8a and GalT-mCherry were treated with either DMSO or GalNAc-*O*-Bn for 20 h. Both DMSO– and GalNAc-*O*-Bn–treated cells were subsequently incubated at 20 °C to accumulate GFP-CD8a in the Golgi before time-lapse imaging at 37 °C in the presence of CHX (see “Experimental procedures”). The images acquired from *D* were quantified and plotted in *E* and *F* as in Fig. 2 (*C* and *D*). In *G* and *H*, the Golgi residence time of TfR under DMSO or GalNAc-*O*-Bn treatment was similarly acquired and plotted as in *E* and *F*. See Fig. S4 (*B* and *C*) for images. In *I* and *J*, after the treatment of DMSO or GalNAc-*O*-Bn for 20 h, cells transiently co-expressing VSVG-SBP-GFP and GalT-mCherry were treated with biotin at 20 °C to accumulate VSVG in the Golgi. Time-lapse imaging was subsequently conducted in the presence of biotin and CHX at 37 °C, and the Golgi residence time was acquired and plotted as *E* and *F*. See Fig. S4 (*D* and *E*) for images. *Scale bar*, 20 μm; *error bar*, S.E.; *p* values are from a *t* test (unpaired and two-tailed); *, *p* ≤ 0.05; **, *p* ≤ 0.005; *NS*, not significant; *n*, number of quantified cells.

We tested two more secretory cargos that possess mucin-type *O*-glycosylation. Resembling Tac, CD8a is a type I transmembrane protein that has a juxtamembrane stem region with multiple *O*-glycosylation sites (Fig. 4*A*) (31). TfR is a type II transmembrane protein with a single *O*-glycosylation site (Thr-104) near its transmembrane domain (Fig. 4*A*). VSVG, a type I transmembrane protein that only undergoes *N*-glycosylation (32), served as a negative control. In cells expressing GFP-CD8a, TfR-GFP, or VSVG-SBP-GFP (in the presence of biotin), we observed that, whereas none appeared at the Golgi in control treatment, CD8a and TfR, but not VSVG, displayed strong Golgi localization under GalNAc-*O*-Bn treatment (Fig. 4 (*B* and *C*) and Fig. S4*A*). Their Golgi residence times under control and GalNAc-*O*-Bn treatment were subsequently measured (Fig. 4 (*D–J*) and Fig. S4 (*B–E*)). We found that GalNAc-*O*-Bn treatment substantially prolonged the Golgi residence times of both CD8a and TfR, but not that of VSVG. Like Tac, we also prepared CD8a with C-terminally or cytosolically tagged GFP (Fig. S4*F*) to rule out the pH and hindrance effect of luminally tagged GFP. The residence times of CD8a-GFP under DMSO or GalNAc-*O*-Bn treatment were very similar to those of an N-terminally tagged one (Fig. S4, *G* and *H*). In summary, our observations suggest that the *O*-glycan might be necessary for the efficient Golgi export of generic *O*-glycosylated cargos.

### The O-glycan is sufficient to promote the Golgi export of Tac(5A)

**Figure 5.**
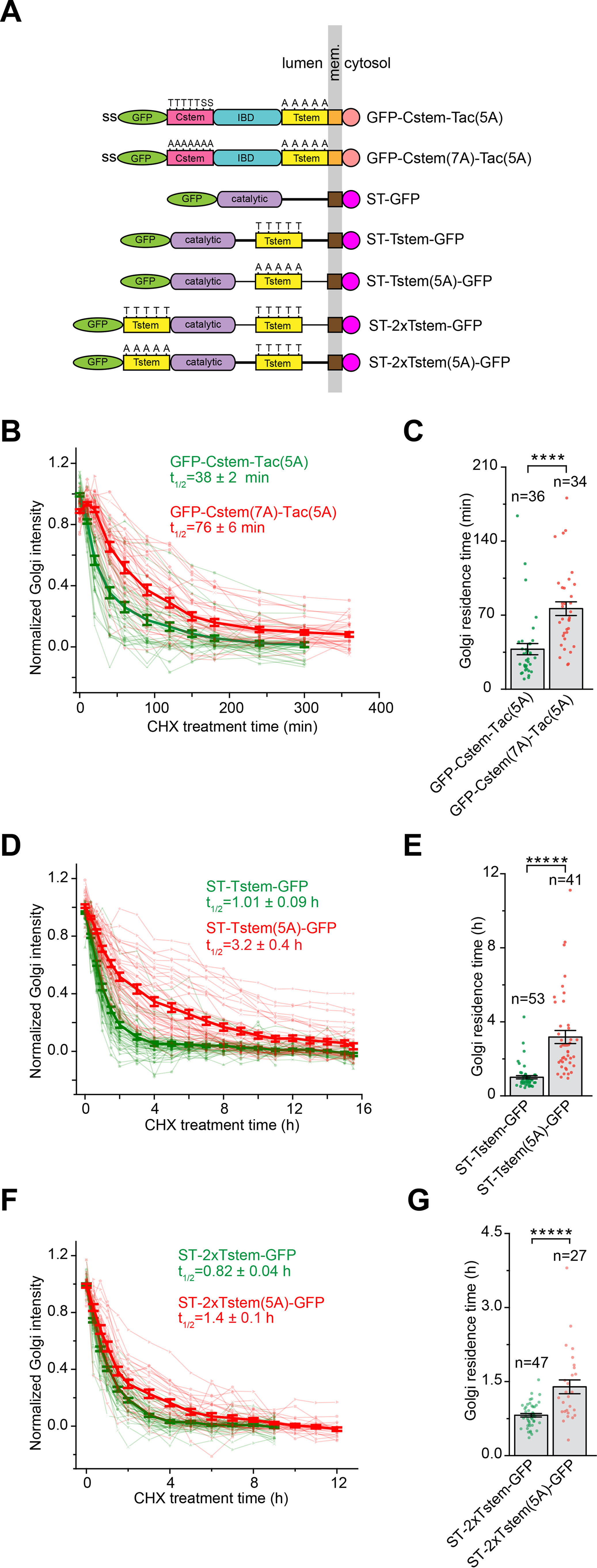
**The *O*-glycan can promote the Golgi export in an additive manner.** *A*, *schematic diagram* showing the domain organization of Tac and ST chimeras. The *panel i*s organized as described for Fig. 1*A*. Thr (*T*) and Ser (*S*) residues that are potentially *O*-glycosylated together with corresponding Ala (*A*) mutations are indicated *above* Cstem or Tstem. *Catalytic*, catalytic domain of ST. *B–G*, Golgi residence times were acquired and plotted, similar to Fig. 2 (*B* and *C*). *B* and *C* demonstrate that introducing the *O*-glycan reduces the Golgi residence time of Tac(5A), therefore suggesting that the *O*-glycan is sufficient to promote the Golgi export. The Golgi residence times shown in *D* and *E* indicate that Tstem in ST-Tstem-GFP are likely *O*-glycosylated. *F* and *G* demonstrate that extra *O*-glycans, introduced by the membrane distal Tstem, promote the efficient Golgi export of ST-2xTstem-GFP compared with ST-2xTstem(5A)-GFP. *Error bar*, mean ± S.E.; *p* values are from a *t* test (unpaired and two-tailed); ****, *p* ≤ 0.00005; *****, *p* ≤ 0.000005; *n*, number of quantified cells.

We investigated the Golgi export of GFP-Tac(5A) (Fig. 3*A*) when *O*-glycans are reintroduced. The stem region of CD8a or Cstem (AAs 134–177) has 5 Thr and 2 Ser residues for potential *O*-glycosylation (Fig. 5*A* and Fig. S5*A*) (31). Cstem was adopted as a transferable sequence for multiple *O*-glycosylation, together with a nonglycosylation negative control, Cstem(7A), in which the above-mentioned Thr and Ser are mutated to Ala. Cstem or Cstem(7A) was subsequently inserted in between GFP and Tac(5A) to make chimeras, GFP-Cstem-Tac(5A) and GFP-Cstem(7A)-Tac(5A) (Fig. 5*A* and Fig. S5*A*). Both chimeras were likely *N*-glycosylated as their gel migration profiles were sensitive to PNGase F treatment (Fig. S5*B*). Furthermore, GFP-Cstem(7A)-Tac(5A) was observed to migrate slightly faster than GFP-Cstem-Tac(5A), consistent with the expectation that the former has fewer or no *O*-glycans. When their Golgi residence times were subsequently measured, we observed that GFP-Tac(5A) with Cstem (38 min) had a much-reduced Golgi residence time compared with that with Cstem(7A) (76 min) (Fig. 5 (*B* and *C*) and Fig. S5*C*), suggesting that the *O*-glycan is probably sufficient to promote the Golgi export of Tac(5A). Altogether, our data demonstrate that the *O*-glycan is both necessary and sufficient for the efficient Golgi export of Tac, therefore supporting our hypothesis that the *O*-glycan might function as a Golgi export signal.

### The O-glycan promotes the Golgi export in an additive manner

We chose ST to study the effect of *O*-glycan's quantity on the Golgi export. We first confirmed that ST is *N*-glycosylated by PNGase F digestion (Fig. S5*D*), as reported previously (33). Although ST is predicted to be *O*-glycosylated in its juxtamembrane sequence (34), we further introduced the Tac stem region (Tstem) (Fig. S5*A*), a known multi-*O*-glycosylation sequence (27), to ST. Tstem or its 5A mutant, Tstem(5A), was inserted into ST's juxtamembrane sequence to make ST-Tstem-GFP or ST-Tstem(5A)-GFP, respectively (Fig. 5*A* and Fig. S5*E*). In these chimeras, the added *O*-glycosylation or mutant sites are proximal to the membrane. Both chimeras were *N*-glycosylated as their gel migration profiles were sensitive to PNGase F treatmentand ST-Tstem(5A)-GFP always migrated slightly faster than ST-Tstem-GFP (Fig. S5*F*), consistent with the prediction that the latter should have more *O*-glycans. When their Golgi residence times were subsequently measured, ST-Tstem-GFP displayed a significantly shorter Golgi residence time (1.01 h) than ST-Tstem(5A)-GFP (3.2 h) (Fig. 5, *D* and *E*), further supporting our finding that *O*-glycosylation promotes the Golgi export.

To introduce more *O*-glycans, an extra copy of Tstem or Tstem(5A), which serves as a negative control, was inserted in between GFP and the catalytic domain of ST to make the chimera ST-2xTstem-GFP or ST-2xTstem(5A)-GFP, respectively (Fig. 5*A* and Fig. S5*E*). In these two chimeras, the newly added *O*-glycosylation and mutant sequences are distal from the membrane. During the subsequent gel analysis, we observed that ST-2xTstem-GFP migrated slower than ST-2xTstem(5A)-GFP (Fig. S5*G*), consistent with the prediction that the former has more *O*-glycosylation sites than the latter. We subsequently measured that the Golgi residence time of ST-2xTstem-GFP (0.82 h) was significantly shorter than that of ST-2xTstem(5A)-GFP (1.4 h). In summary, our observation that a cargo with more *O*-glycans can have a shorter Golgi residence time implies that the *O*-glycan might promote the Golgi export in an additive manner.

### The N-glycan might also function as a Golgi export signal

**Figure 6.**
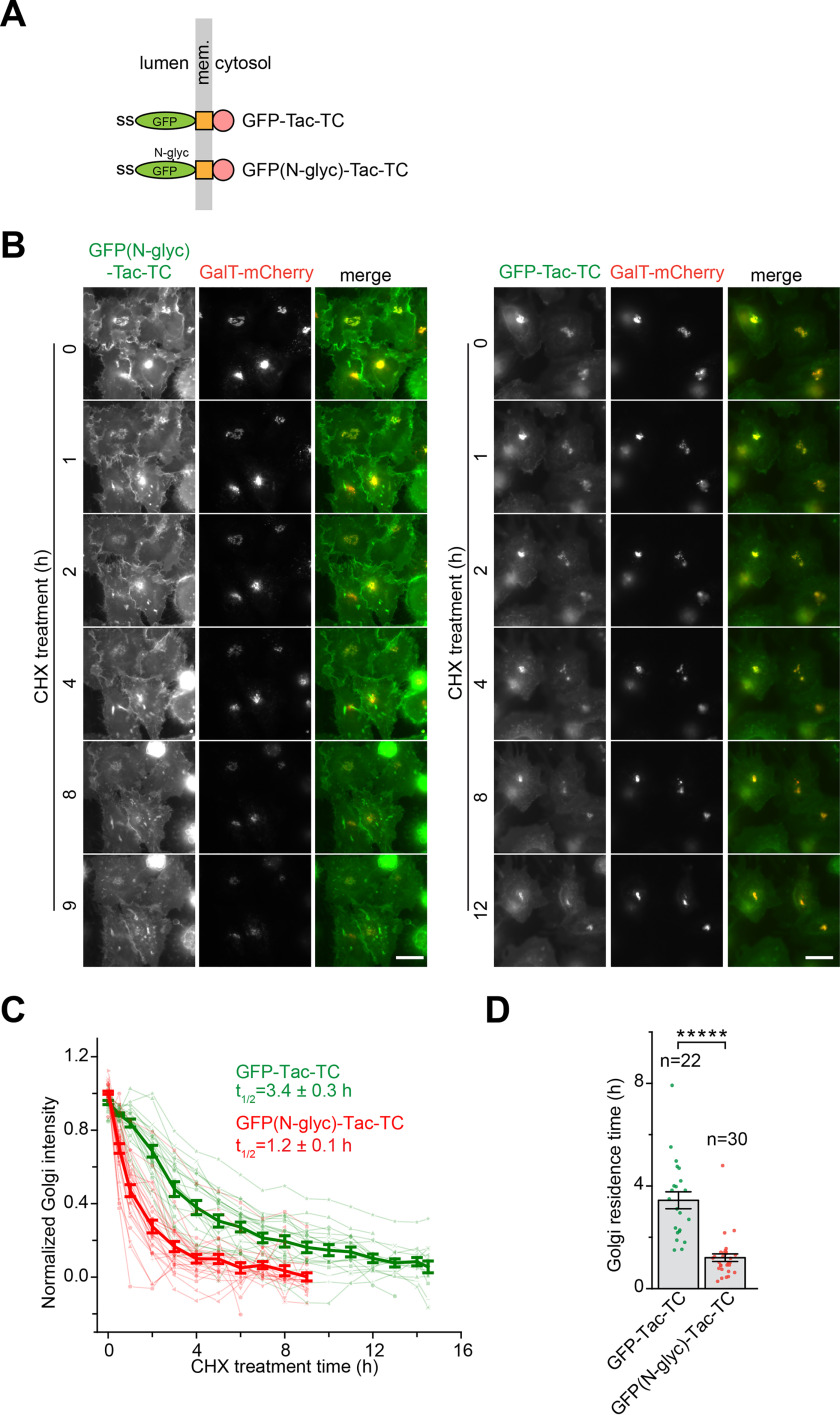
**The *N*-glycan can accelerate the Golgi export of GFP-Tac-TC; HeLa cells were used.** *A*, schematic diagram showing the domain organization of Tac-TC fused to *N*-glycosylated (*N-glyc*) or WT GFP. The *panel i*s organized as described for Fig. 1*A*. *B–D*, introduction of the *N*-glycan significantly promotes the Golgi export of GFP-Tac-TC. The experiment and *panel o*rganization are similar to those of Fig. 2 (*B–D*). GFP-Tac-TC data are from Fig. 2*C*. *Scale bar*, 20 μm; *error bar*, mean ± S.E.; *p* values are from a *t* test (unpaired and two-tailed); *****, *p* ≤ 0.000005; *n*, number of quantified cells.

We asked whether the *N*-glycan can also function as a Golgi export signal. Whereas the *O*-glycan is mostly found in the unfolded or disordered regions, such as interdomain linkers or loops, the *N*-glycosylation mostly occurs in protein domains and is essential for the proper folding of a protein in the ER (34). To circumvent the requirement of the *N*-glycosylation in the folding and subsequent ER export of cargos, we chose the *N*-glycosylated GFP because GFP can fold in the ER without the requirement of glycosylation. Missing the luminal region of Tac, GFP-Tac-TC does not undergo *N*-glycosylation. A sequon *N*-glycosylation site was introduced to a loop connecting β6 and β7 of GFP to make GFP(*N*-glyc) (35), and GFP(*N*-glyc)–tagged Tac-TC was constructed (Fig. 6*A*). In the subsequent gel analysis, we observed that GFP(*N*-glyc)-Tac-TC migrated slower than GFP-Tac-TC, and, after PNGase F treatment, both chimeras migrated at the same speed (Fig. S6), demonstrating the successful *N*-glycosylation of GFP(*N*-glyc)-Tac-TC. Our live-imaging data showed that the Golgi residence time of GFP(*N*-glyc)-Tac-TC (1.2 h) was reduced to almost one-third that of GFP-Tac-TC (3.4 h) (Fig. 6, *B–D*), therefore suggesting that the *N*-glycan might function as a Golgi export signal, similar to what we found for the *O*-glycan.

### Tac-TC accumulates at the interior of the trans-Golgi cisternae

**Figure 7.**
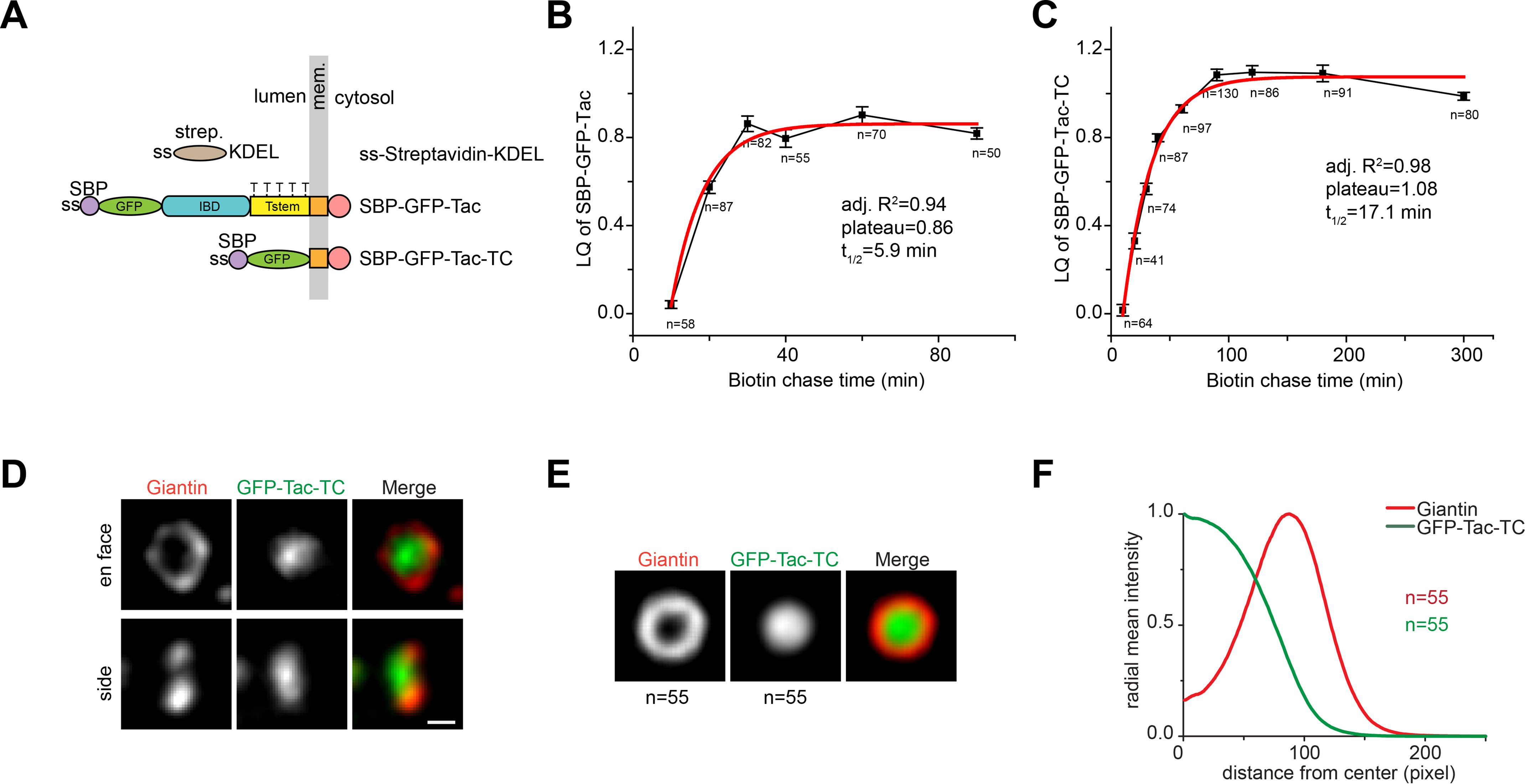
**Tac-TC accumulates at the interior of the *trans*-Golgi cisternae; HeLa cells were used.** *A*, schematic diagram showing the domain organization of Tac and Tac-TC RUSH reporters. The *panel i*s organized as described for Fig. 1*A*. ss-Streptavidin-KDEL is the ER hook. *Strep*., streptavidin. The 5 Thr residues that are potentially under mucin-type *O*-glycosylation are indicated on Tstem. *B* and *C*, intra-Golgi transport kinetics of Tac and Tac-TC as revealed by the LQ *versus* time plot. Cells transiently co-expressing the RUSH reporter, SBP-GFP-Tac or SBP-GFP-Tac-TC, and GalT-mCherry were treated with nocodazole to induce the formation of Golgi mini-stacks. The ER-arrested RUSH reporter was subsequently released by the administration of biotin. At different time points, cells were immunostained for endogenous GM130, and the LQs for the RUSH reporter were acquired and fitted to the first-order exponential function. *Error bars*, mean ± S.E.; *n*, number of Golgi mini-stacks quantified. *Adj. R^2^*, adjusted R^2^. *D–F*, Tac-TC localizes to the interior of Golgi cisternae at the steady state. *D*, Airyscan superresolution images of the *en face* and *side view* of giantin and GFP-Tac-TC in the Golgi mini-stack. Cells transiently expressing GFP-Tac-TC were treated with nocodazole to induce the formation of Golgi mini-stacks and stained for endogenous giantin. The *en face* and *side view* images were selected. *Scale bar*, 500 nm. The *en face* averaging of giantin and GFP-Tac-TC in the Golgi mini-stack is shown in *E*. The corresponding radial mean intensity profile is displayed in *F*, with the distance from the center of fluorescence mass (giantin peak is normalized to 100) as the *x* axis and the radial mean intensity (normalized) as the *y* axis. *n*, number of Golgi mini-stack images used.

To compare the intra-Golgi transport of Tac-TC with Tac, corresponding RUSH reporters were constructed (Fig. 7*A*). During the chase, the synchronized trafficking of Tac-TC and Tac was quantitatively monitored by the localization quotient (LQ), a linear metric of the axial Golgi localization, using our recently developed Golgi superresolution tool, GLIM (Golgi protein localization by imaging centers of mass) (36). Both Tac-TC and Tac rapidly entered the secretory pathway from the ER and transited from the *cis*- to *trans*-side of the Golgi, as shown by increases of their LQs (Fig. 7, *B* and *C*). *t*_½_ values from the first-order exponential fitting of the LQ *versus* time plots reflect the intra-Golgi transport velocity. Similar to their Golgi export, the intra-Golgi transport velocity of Tac-TC (*t*_½_ = 17.1 min) is slower than that of Tac (*t*_½_ = 5.9 min). Like Tac (Fig. 7*B*) and other constitutive secretory cargos that we studied previously (36), the LQ of Tac-TC plateaued at ∼1 (Fig. 7*C*), a localization roughly corresponding to the *trans*-Golgi (36). Consistently, steady-state LQs of Tac-TC and Tac(5A), which were acquired from cells expressing respective GFP-tagged construct, were measured to be 0.94 ± 0.02 (*n* = 118) and 0.95 ± 0.02 (*n* = 123). The observation that the slow Golgi exit cargo accumulates at the *trans*-Golgi provides further evidence that the *trans*-Golgi instead of the TGN is the export site of constitutive secretory cargos. By identifying *en face* views of nocodazole-induced Golgi mini-stacks (37), we found that the *trans*-localized Tac-TC and Tac(5A) mainly distribute to the interior of Golgi cisternae under Airyscan superresolution microscopy (Fig. 7 (*D–F*) and Fig. S7), similar to other secretory cargos that we studied previously (37).

## Discussion

The role of glycans in the secretory trafficking is still unclear. Previously, Gut *et al.* (17) and Proszynski *et al.* (18) qualitatively demonstrated that the glycan can be a determinant for the Golgi-to-PM trafficking. Here, by quantitatively measuring the Golgi residence times of transmembrane secretory reporters, we further pinpointed that both *O*- and *N*-glycans can function as a signal for the efficient Golgi export. Abolishing or inhibiting the *O*-glycosylation of Tac, CD8a, and TfR by mutagenesis or small molecule substantially slowed down their Golgi export and resulted in their Golgi localization or retention, whereas adding *O*-glycans to Golgi-localized Tac(5A) and ST produced the opposite effect (*i.e.* the promotion of their Golgi export and the reduction of their Golgi retention). Similarly, introducing the *N*-glycan to GFP also accelerated the Golgi export of the engineered reporter, GFP-Tac-TC. Using the conventional Golgi resident ST as a reporter, we demonstrated that the Golgi export promotion effect of the *O*-glycan can be additive. Therefore, glycans can be necessary and sufficient for the efficient Golgi export, and our discovery might be extended to generic transmembrane proteins, including conventional Golgi residents and transiting secretory cargos. It has been reported previously that the apical targeting of cargos is compromised when their *N*- or *O*-glycosylation is disrupted (15, 16). Retrospectively and considering our current finding, the compromised apical targeting is likely due to the slow Golgi export of glycan-deficient cargos. The *O*-glycosylation at the stem region has also been documented to protect cell surface proteins, such as low-density lipoprotein receptor (38), TfR (39), decay-accelerating factor (40), and Tac (41), from ectodomain shedding. Therefore, our discovery further expands the role of the *O*-glycan in the expression of cell surface receptors by suggesting that it contributes to their Golgi-to-PM or exocytic trafficking as well.

Most protein trafficking signals are cytosolic AA sequences, which are recognized by trafficking machinery components, such as vesicular coats and adaptors. Hence, as a Golgi export signal, the glycan is unique because it is a nonproteinaceous moiety and confined to the lumen. Probably, it makes biological sense for the glycan to function as a Golgi export signal as it ensures sufficient glycosylation of secretory cargos before leaving the Golgi. What type of sorting information does the glycan encode? And how is it recognized and recruited to an exocytic carrier by the trafficking machinery? It has been previously postulated that glycans might interact with lipid rafts or lectins such as VIP36 for their roles in the apical targeting (15, 16). We argue that, different from conventional trafficking signals, glycans except M6P (11, 12) are unlikely to be sorted by a receptor-mediated mechanism due to their vast diversity in structure and composition. Instead, we speculate that their sorting is primarily based on physico-chemical properties of the glycan, such as the size, shape, charge (potentially contributed by sialic acids), and hydrophilicity.

Although other mechanisms are certainly possible, our data are compatible with a simple model in which the sorting of glycans to exocytic carriers is by the molecular exclusion mechanism, which is elaborated below. Previous imaging data have demonstrated that the interior of the medial and *trans*-Golgi cisternae is tightly packed with arrays of Golgi glycosylation enzymes (42, 43), which constitute what we termed “the Golgi enzyme matrix.” During the cisternal transition of a secretory cargo from the *cis*- to *trans*-side of the Golgi, the cargo mainly partitions to the Golgi enzyme matrix (37). In this process, the addition of glycans increases the hydrodynamic volume or hydrophilicity of the cargo's luminal region, probably resulting in its exclusion from the enzyme matrix. Hence, the expelled but fully glycosylated cargo ends up in the rim of Golgi cisternae, where trafficking machinery components subsequently pack it into the exocytic carrier (37). Using the analogy of the gel filtration column, a non- or underglycosylated cargo probably has a small Stokes radius and therefore a large elution volume or long Golgi residence time, whereas the glycosylated cargo has the opposite as a result of its increased size. Especially, for GPI-anchored proteins, the juxtamembrane glycan moiety might directly serve as a Golgi export signal. For the nonglycosylated cargos, which are a minority, other mechanisms are probably utilized to facilitate their efficient Golgi export. For example, multimerization may increase the hydrodynamic volume so that cargos are excluded from the interior of the Golgi enzyme matrix. Indeed, we previously demonstrated that large soluble secretory proteins always localize to the rim of Golgi cisternae during their intra-Golgi transport (37). Further experiments are certainly required to test this view of the Golgi export.

## Experimental procedures

### DNA plasmids

See Table S1. The following DNA plasmids were described previously: Tac-GFP-intermediate (44), CD8a-GFP (44), CD8a (45), GFP-GM130 (46), GalT-mCherry (47), Sec61β-mCherry (36), CD8a-furin-mEOS2 (48), pET30ax (49), SBP-mCherry-GPI (25), TNFα-SBP-GFP (25), VSVG-SBP-GFP (25), SBP-GFP-E-cadherin (25), and GFP-ERGIC53 (50).

### Antibodies, enzyme, and small molecules

Mouse anti-Lamp1 mAb (H4A3; 1:500 for immunofluorescence or hereafter IF), mouse anti-CD63 mAb (H5C6; 1:100 for IF), and mouse anti-CD8a mAb (OKT8; 1:500 for IF) were from the Developmental Studies Hybridoma Bank. Mouse anti-GFP mAb (catalog no. sc-9996; 1:1000 for Western blotting) was purchased from Santa Cruz Biotechnology, Inc. Mouse anti-EEA1 mAb (catalog no. 610456; 1:500 for IF) and mouse anti-GM130 mAb (catalog no. 610823; 1:500 for IF) were from BD Biosciences. Horseradish peroxidase–conjugated goat anti-mouse (catalog no. 176516; 1:10,000 for Western blotting) was from Bio-Rad. Alexa Fluor–conjugated goat antibodies against mouse IgG (1:500 for IF) were from Thermo Fisher Scientific.

PNGase F was purchased from New England Biolabs. The following small molecules were commercially available: nocodazole (Merck; working concentration: 33 μm), brefeldin A (Epicenter Technologies; working concentration: 10 µg/ml), cycloheximide (Sigma–Aldrich; working concentration: 10 µg/ml), and GalNAc-*O*-Bn (Sigma–Aldrich; working concentration: 2 mm).

### Production of rabbit anti-mCherry polyclonal antibody

The plasmid DNA encoding His-mCherry (mCherry-pET30ax) was used to transform BL21DE3 *Escherichia coli* cells, and resulting bacteria were induced by 0.25 mm isopropyl β-d-thiogalactopyranoside (IPTG) (Thermo Fisher Scientific) at 16 °C overnight. Bacterial cells were pelleted by centrifugation and lysed by sonication in PBS with 8 m urea. The supernatant was collected by centrifugation and incubated with nickel-nitrilotriacetic acid agarose beads at room temperature for 2 h. The beads were washed by PBS supplemented with 8 m urea and 20 mm imidazole and eluted using PBS supplemented with 8 m urea and 250 mm imidazole. The eluted His-mCherry was concentrated, and the buffer was changed to PBS containing 4 m urea. His-mCherrry was used as an antigen to immunize rabbits, and antisera were collected by Genemed Synthesis Inc.

The plasmid DNA encoding GST-mCherry (mCherry-pGEB) was used to transform BL21DE3 *E. coli* cells. Bacterial cells were induced by 0.25 mm IPTG at 16 °C overnight and collected by centrifugation, followed by sonication in the lysis buffer containing 50 mm Tris, pH 8.0, 100 mm NaCl, 0.1% Triton X-100, and 1 mm DTT (Sigma–Aldrich). After centrifugation, the collected supernatant was incubated with GSH-Sepharose beads (GE Healthcare) at 4 °C overnight and washed by the lysis buffer. After further washing by 200 mm sodium borate buffer (pH 9.0), GST-mCherry immobilized on beads was cross-linked to beads by incubating with sodium borate buffer supplemented with 50 mm dimethyl pimelimidate (Sigma–Aldrich), followed by neutralization with 200 mm ethanolamine (pH 8.0). Cross-linked beads were incubated with mCherry antisera for 1 h at room temperature. After PBS washing, the bound mCherry antibody was eluted by 100 mm glycine, pH 2.8, neutralized by 1 m Tris, pH 8.0, and dialyzed in PBS.

### Cell culture and transfection

HeLa cells were cultured in Dulbecco's modified Eagle's medium supplemented with 10% fetal bovine serum (FBS). For IF labeling, cells were seeded onto a Φ 12-mm glass coverslip in a 24-well plate. For fluorescence live imaging, cells were seeded onto a Φ 35-mm glass-bottom Petri dish (Cellvis) and imaged in the CO_2_-independent medium (Thermo Fisher Scientific) supplemented with 4 mm glutamine and 10% FBS at 37 °C. Lipofectamine 2000 (Thermo Fisher Scientific) was used to transfect cells according to standard protocol from the manufacturer. Experiments were conducted ∼20 h after transfection.

### IF labeling

Cells grown on Φ 12-mm glass coverslips were fixed by 4% paraformaldehyde in PBS. After washing, cells were incubated with 100 mm NH_4_Cl to block the residual paraformaldehyde and subjected to further wash in PBS. Next, cells were sequentially incubated with the primary antibody followed by the secondary antibody (Alexa Fluor–conjugated goat anti-mouse and/or rabbit antibodies) diluted in PBS containing 5% FBS, 2% BSA, and 0.1% saponin (Sigma–Aldrich). A washing step was included between the primary and secondary antibody incubation. After extensive wash in PBS, labeled cells were mounted in Mowiol mounting medium, containing 12% Mowiol 4-88 (EMD Millipore), 30% glycerol, and 100 mm Tris, pH 8.5. Once dried, coverslips were sealed in nail polish.

### Conventional fluorescence microscopy

Live and fixed cells were imaged under Olympus IX83 inverted wide-field microscope system equipped with an oil ×100/NA 1.40 objective (Plan Apo), an oil ×63/NA 1.40 objective (Plan Apo), and an oil ×40/NA 1.20 objective (fluorite), a motorized stage, a focus drift correction device, a 37 °C enclosed environment chamber, motorized filter cubes, a scientific complementary metal oxide semiconductor camera (Neo; Andor), and a 200-W metal-halide excitation light source (Lumen Pro 200; Prior Scientific). Filters and dichroic mirrors were optimized for GFP/Alexa Fluor 488, mCherry/Alexa Fluor 594, and Alexa Fluor 680. The microscope system was controlled by MetaMorph (Molecular Devices).

### Superresolution fluorescence microscopy

This was conducted using Airyscan superresolution microscope system (Carl Zeiss) as described previously (37).

### Measuring LQs

This was described previously (36, 43).

### Golgi residence time quantification

HeLa cells grown on a Φ 35-mm glass-bottom Petri dish were co-transfected with a fluorescence protein–tagged Golgi marker, GalT-mCherry or GFP-GM130, together with the indicated GFP- or mCherry-tagged reporter for about 20 h. For reporters that have substantial steady-state Golgi localization, cells were directly subjected to live imaging in the CO_2_-independent medium (Thermo Fisher Scientific) supplemented with 10% FBS, 4 mm glutamine, and 10 µg/ml CHX at 37 °C. At the steady state, GFP-Tac, GFP-CD8a, and TfR-GFP do not have significant Golgi localization. To accumulate or synchronize these reporters in the Golgi, cells expressing them were incubated at 20 °C for 3 h in the absence of CHX followed by 20 °C for 1 h before live imaging at 37 °C in the presence of CHX. For RUSH reporters, cells expressing them were first incubated with 50 ng/ml streptavidin to quench the residual amount of biotin present in the tissue culture medium. After 20 h, cells were washed extensively to remove the streptavidin and incubated with 40 μm biotin and 10 µg/ml CHX at 20 °C to accumulate RUSH reporters in the Golgi before live imaging at 37 °C.

For each reporter, 2D time-lapse imaging was conducted until the reporter fluorescence signal in the Golgi almost disappeared. Typically, <20 frames were acquired during each time-lapse imaging, and therefore the photobleaching was negligible. Golgi residence times were subsequently quantified from these imaging data using ImageJ (National Institutes of Health). In a time lapse, Golgi regions of interest (ROIs) were generated by the intensity segmentation of co-expressed GalT-mCherry or -GFP with manual modifications. The total Golgi intensity of the reporter at each time point was measured in ImageJ and fitted by the first-order exponential decay function, *y* = *y*_0_ + A_1_ exp(−(*x* − *x*_0_)/*t*_1_), in OriginPro8.5 (OriginLab). The Golgi residence time, which is defined as the *t*_½_, was then calculated as 0.693 × *t*_1_. Only time lapses with adjusted *R*^2^ ≥ 0.80 and the length of acquisition ≥1.33 × *t*_½_ were considered in our calculation.

### Quantifying the Golgi-to-cell intensity ratio

The analysis was conducted in ImageJ. The images acquired in Fig. 1*B* were first background-subtracted. Golgi ROIs were generated by the intensity segmentation of co-stained GM130 signal, whereas cell ROIs were manually drawn. The Golgi-to-cell intensity ratio of a cell was calculated as the ratio of the intensity of the Golgi ROI to that of the cell ROI.

### PNGase F digestion

HeLa cells seeded in a 24-well plate were transiently transfected with the reporter DNA plasmid. After centrifugation, pellets were suspended in 10 µl of glycoprotein denaturing buffer containing 0.5% SDS and 40 mm DTT and heated at 100 °C for 10 min to denature proteins. Next, 2 µl of GlycoBuffter 2 (10×, 500 mm sodium phosphate, pH 7.5), 2 µl of 10% Nonidet P-40, 6 µl of H_2_O, and 1 µl of PNGase F (New England Biolabs) were added into the cell lysate, and the mixture was incubated at 37 °C for 1 h. After adding SDS-sample buffer, the digested cell lysate was analyzed by standard SDS-PAGE followed by Western blotting. Western blotting and molecular weight marker bands were imaged under the chemiluminescence and white light mode, respectively, using LAS-4000 (GE Healthcare Life Sciences). Uncropped blot images are presented in Fig. S8.

### GalNAc-O-Bn treatment

HeLa cells transiently expressing the reporter of interest were incubated with the tissue culture medium containing 2 mm GalNAc-*O*-Bn or DMSO as a control for 20 h before fluorescence imaging or immunoblotting.

### VHH (variable heavy-chain domain of heavy-chain-only antibody)-mCherry purification

His_6_-tagged VHH-mCherry (Addgene, catalog no. 109421) was transformed into BL21DE3 *E.coli* cells. Bacterial culture was induced by 0.25 mm IPTG at 16 °C overnight. Cells were harvested by centrifugation and resuspended using the lysis buffer (100 mm HEPES, pH 7.4, 500 mm KCl, 1% Triton X-100, 200 μg/ml lysozyme, 0.5 mm phenylmethylsulfonyl fluoride, 10 mm imidazole, and 2 mm DTT), followed by sonication lysis. After centrifugation, the resulting supernatant was incubated with nickel-nitrilotriacetic acid agarose beads (Qiagen) pre-equilibrated with the washing buffer (20 mm HEPES, pH 7.4, 200 mm KCl, 10% glycerol, and 2 mm DTT) supplemented with 10 mm imidazole at 4 °C overnight. Beads were then washed by the washing buffer supplemented with 25 mm imidazole and eluted by the washing buffer supplemented with 250 mm imidazole. The elute was dialyzed in PBS at 4 °C overnight and quantified by Coomassie Blue–stained SDS-PAGE using BSA as a standard.

### Cell internalization assay

HeLa cells transiently expressing the indicated GFP-tagged protein, CD8a-furin-mEOS2, or SBP-mCherry-GPI were incubated with 5 µg/ml VHH-mCherry, mouse anti-CD8a mAb, or rabbit anti-mCherry polyclonal antibody, respectively, for 2 h, followed by fixation and immunostaining for the indicated proteins.

## Data availability

All data are included in the article and the supporting information.

## Supplementary Material

Supporting Information
