# Supplementary material for: Glycans function as a Golgi export signal to promote the constitutive exocytic trafficking
Source: J Biol Chem. 2020 Aug 21;295(43):14750–62. doi: 10.1074/jbc.RA120.014476 (PMC7586228; doi:10.1074/jbc.RA120.014476)
Supplement: Supporting Information [file supp_295_43_14750__index.html]

Glycans function as a Golgi export signal to promote the constitutive exocytic trafficking — Glycans promote the Golgi export — Glycans function as a Golgi export signal to promote the constitutive exocytic trafficking — Glycans promote Golgi export — Supporting Information 

# Glycans function as a Golgi export signal to promote the constitutive exocytic trafficking

## Supporting Information

- Supporting Information (to be published online) - Figure S1-8 and Table S1
