# Supplementary material for: Glycans function as a Golgi export signal to promote the constitutive exocytic trafficking
Source: J Biol Chem. 2020 Aug 21;295(43):14750–62. doi: 10.1074/jbc.RA120.014476 (PMC7586228; doi:10.1074/jbc.RA120.014476)
Supplement: Supporting Information [file supp_RA120.014476_161006_2_supp_583009_qf8mvy.pdf]

Supporting Information

Glycans function as a Golgi export signal to promote the constitutive exocytic trafficking

Xiuping Sun[1], Hieng Chiong Tie[1], Bing Chen[1] and Lei Lu[1,*]

[1]School of Biological Sciences, Nanyang Technological University, 60 Nanyang Drive, Singapore 637551.

*Correspondence should be addressed to:
Lei Lu (PhD), School of Biological Sciences, Nanyang Technological University, 60 Nanyang Drive, Singapore 637551; Tel: 65-65922591; Fax: 65-67913856; Email: lulei@ntu.edu.sg

File contents:

Figure S1-8

Table S1

Figure S1

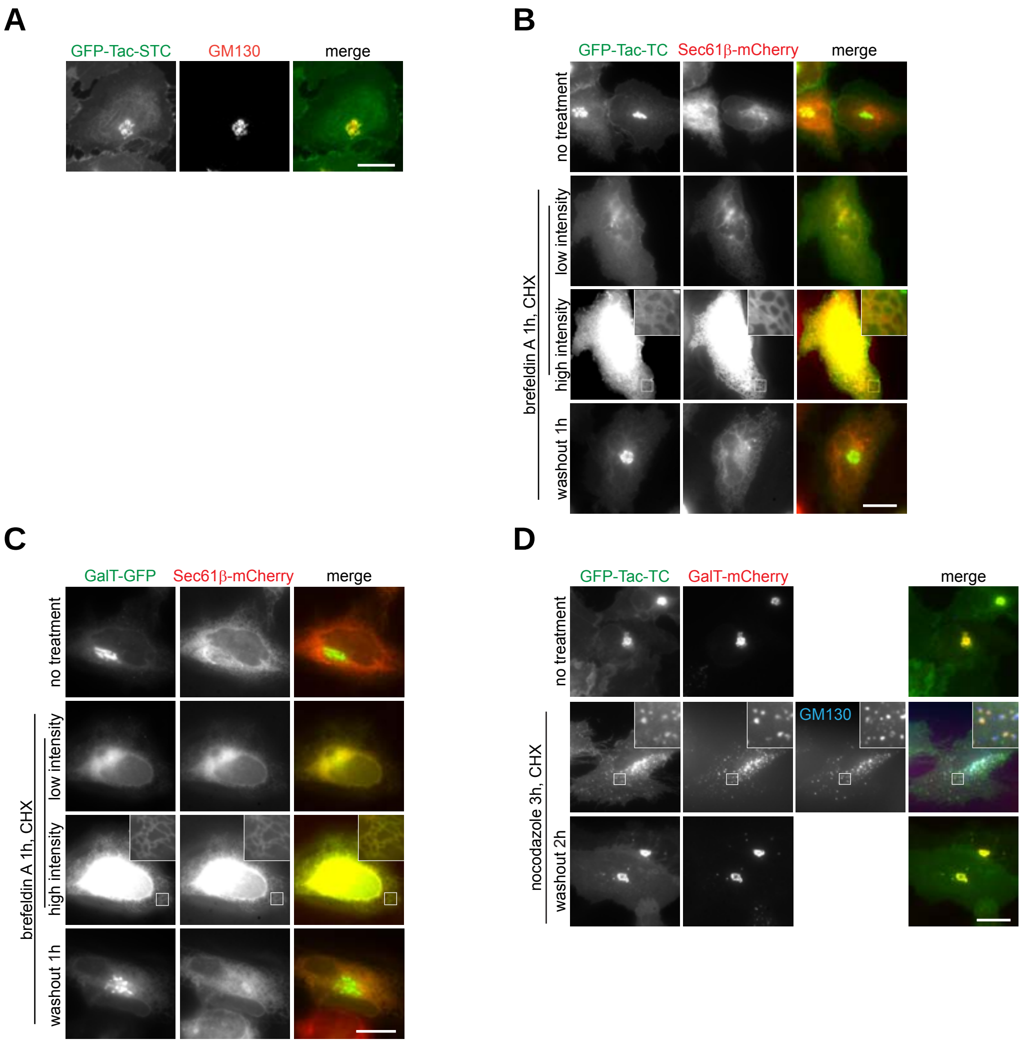

Figure S1

Tac-TC behaves like a Golgi resident. HeLa cells were used. (A) Example image showing the Golgi localization of GFP-Tac-STC. Cells transiently expressing GFP-Tac-STC were immuno-stained for endogenous GM130. (B,C) Tac-TC and GalT localize to the ER under brefeldin A treatment and to the recovered Golgi during the subsequent washout. Cells transiently co-expressing Sec61β-mCherry and GFP-Tac-TC (B) or GalT-GFP (C) were subjected to the indicated treatment and imaged live. The same images with different intensity scaling are shown in the second and third row to reveal the weak ER localization signal. (D) Tac-TC and GalT localize to the Golgi mini-stack under nocodazole treatment and to the recovered Golgi during the subsequent washout. Cells transiently co-expressing GFP-Tac-TC and GalT-mCherry were subjected to the indicated treatment and subsequently immuno-stained for endogenous GM130 (for images in the second row). In (B-D), boxed regions are enlarged at the upper right corner; scale bar, 20 μm.

Figure S2

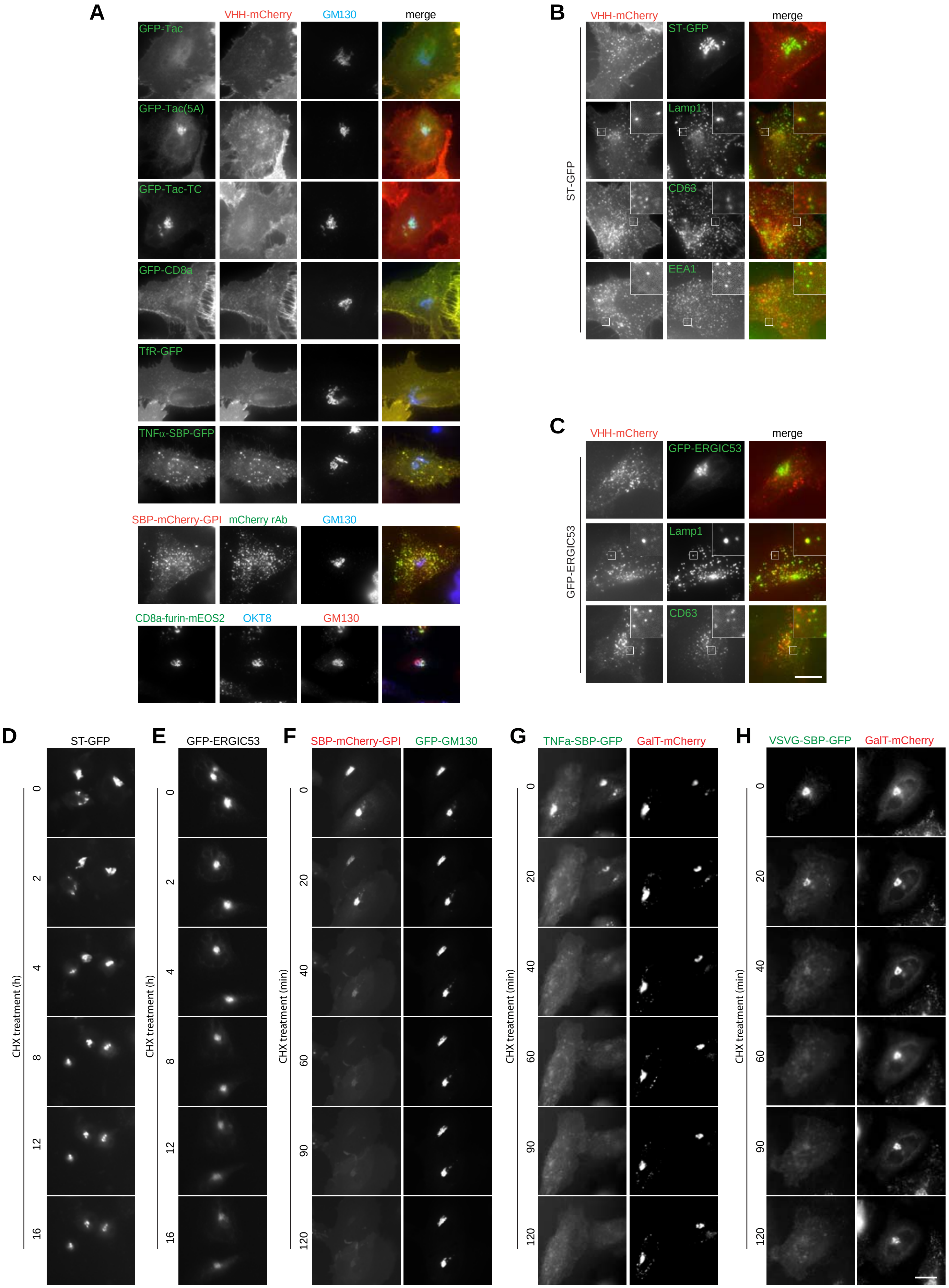

A
GFP-Tac
VHH-mCherry
GM130
merge
GFP-Tac(5A)
GFP-Tac-TC
GFP-CD8a
TfR-GFP
TNFα-SBP-GFP
SBP-mCherry-GPI
mCherry rAb
CD8a-furin-mEOS2
OKT8
GM130
B
VHH-mCherry
ST-GFP
merge
Lamp1
CD63
EEA1
C
GFP-ERGIC53
D
CHX treatment (h)
0
2
4
8
12
16
E
F
SBP-mCherry-GPI
GFP-GM130
CHX treatment (min)
0
20
40
60
90
120
G
TNFa-SBP-GFP
GalT-mCherry
H
VSVG-SBP-GFP

Figure S2

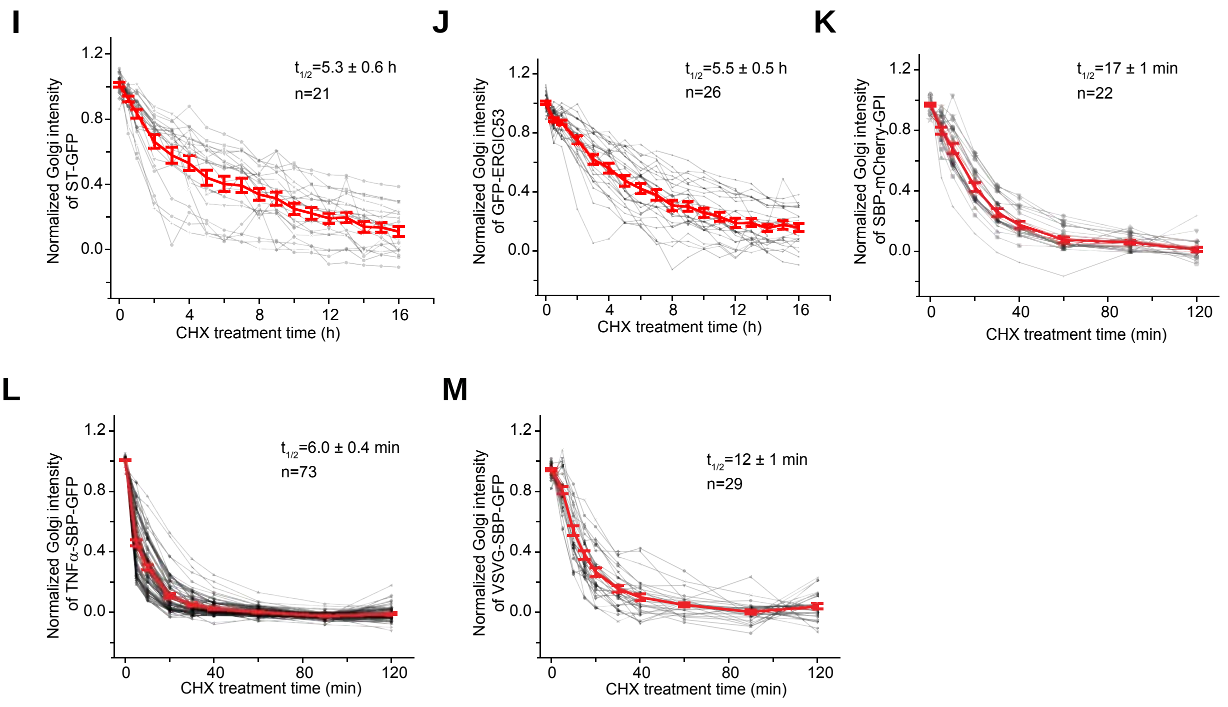

Figure S2

Investigating the endocytic trafficking and Golgi residence times of secretory cargos and Golgi residents. HeLa cells were used. (A-C) Reporters employed in this study do not target to the Golgi from the PM or endolysosome. In (A), cells transiently expressing indicated fluorescence protein-tagged reporter were incubated with VHH-mCherry, rabbit anti-mCherry polyclonal antibody or mouse anti-CD8a monoclonal antibody for 2 h and subsequently subjected to immuno-staining of endogenous GM130 and the internalized antibody. CD8a-furin-mEOS2 was a positive control for the Golgi targeting from the PM or endolysosome. In (B,C), surface-localized ST-GFP and GFP-ERGIC53 are targeted to the endolysosome instead of the Golgi. Cells transiently expressing ST-GFP (B) or GFP-ERGIC53 (C) were incubated with VHH-mCherry for 2 h and subsequently subjected to immuno-staining of endogenous Lamp1 (a lysosome marker), CD63 (a late endosome marker) or EEA1 (an early endosome marker). Boxed regions were enlarged at the upper right corner. (D-M) Acquiring the Golgi residence times of Golgi residents and secretory cargos. These panels are related to Figure 2D. In (D-H), cells expressing indicated fluorescence protein-tagged reporter(s) were imaged live in the presence of CHX. Although not shown, GalT-mCherry was also co-expressed in (D,E). In (I-M), the corresponding total Golgi intensity was plotted as described in Figure 2C. Scale bar, 20 µm; error bar, mean ± standard error; n, the number of quantified cells.

Figure S3

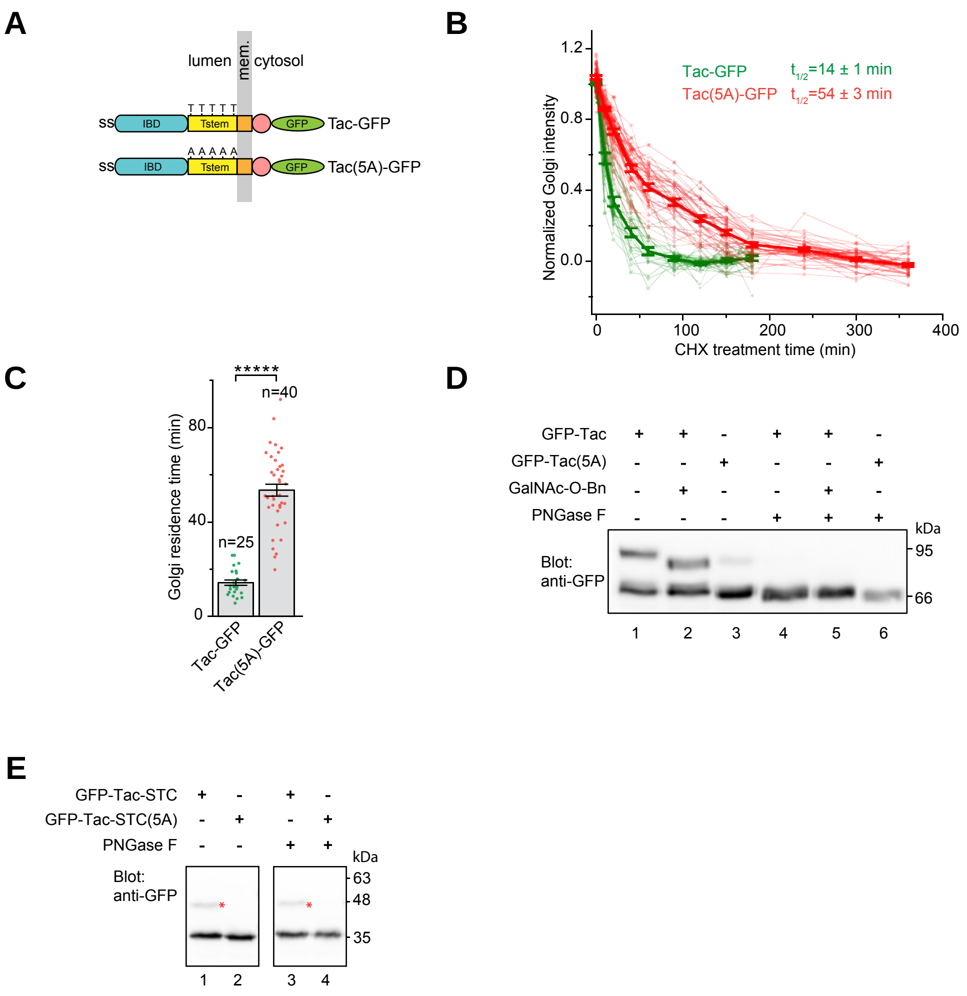

Figure S3

The Golgi residence times and gel migration profiles of Tac mutants. (A) The schematic diagram showing the domain organization and glycosylation or mutation sites of C-terminally GFP-tagged Tac and Tac(5A). The panel is organized as described in Figure 3A. (B,C) The Golgi residence time of Tac(5A)-GFP is significantly longer than that of Tac-GFP. The experiment and panel organization are described in Figure 3, B-E. Error bar, mean ± standard error; *P* values are from *t* test (unpaired and two-tailed); *****, $P \leq 0.000005$; n, the number of quantified cells. (D) GalNAc-O-Bn inhibits the O-glycosylation of Tac. HeLa cells transiently expressing GFP-Tac or GFP-Tac(5A) were treated or not with GalNAc-O-Bn for 20 h. Cell lysates were further treated or not with PNGase F before being immuno-blotted for GFP-tag. In comparison with lane 1, the disappearance of the upper band and the slightly increased migration of the lower band in lane 4 imply that the two bands of GFP-Tac in lane 1 are N-glycosylated. Likewise, lane 1 and 3 suggest that the two bands of GFP-Tac in lane 1 are O-glycosylated. The increased migration of the upper band in lane 2 compared with lane 1 indicates that GalNAc-O-Bn probably inhibits the O-glycosylation of Tac. (E) Only a small fraction of Tac-STC is O-glycosylated. The lysate of HeLa cells transiently expressing GFP-Tac-STC or GFP-Tac-STC(5A) was treated or not with PNGase F before immune-blotted for GFP-tag. * indicates the O-glycosylated band. The left and right panels are cropped from the same gel blot. 1-4 indicate lanes of the gel. Molecular weight markers (kDa) are indicated at the right.

Figure S4

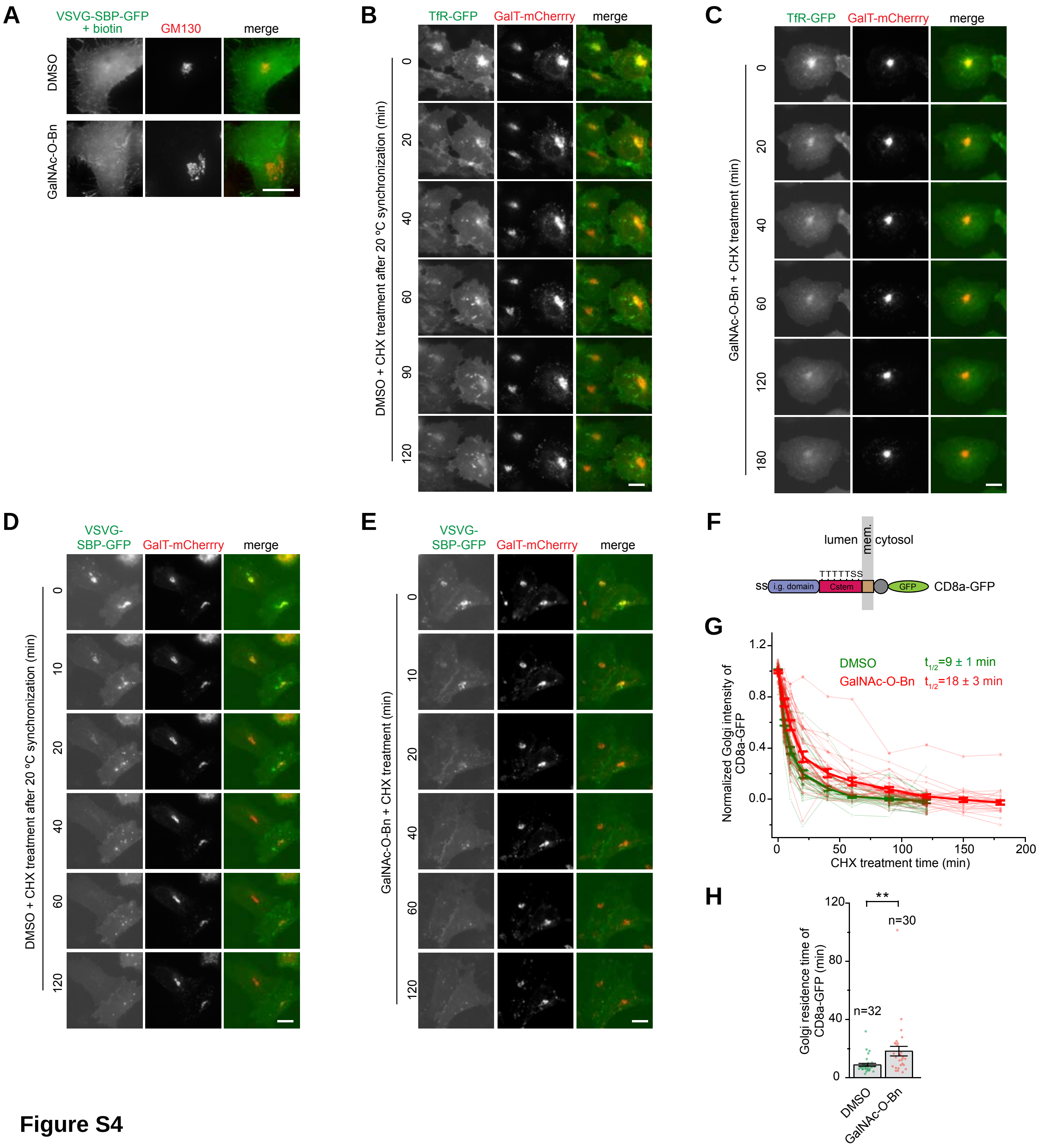

Figure S4

The effect of GalNAc-O-Bn treatment on the Golgi localization of TfR and VSVG and the Golgi residence times of CD8a-GFP. HeLa cells were used. (A-E) Inhibiting the O-glycosylation results in the substantial Golgi localization of TfR but not VSVG. (A) After the treatment of DMSO or GalNAc-O-Bn, cells transiently expressing VSVG-SBP-GFP were further treated with biotin for 14 h before immuno-staining of endogenous GM130. (B,C) Cells transiently co-expressing TfR-GFP and GalT-mCherry were subjected to similar experimental procedure as described in Figure 4D. (D,E) As described in Figure 4, I and J. Scale bar, 20 µm. (F-H) GalNAc-O-Bn treatment substantially increases the Golgi residence time of C-terminally GFP-tagged CD8a. (F) The schematic diagram showing the domain organization and potential O-glycosylation sites of CD8a-GFP. The panel is organized as described in Figure 4A. (G,H) The Golgi residence times of CD8a-GFP under DMSO or GalNAc-O-Bn treatment. The experiment and panel organization are the same as those of Figure 4, D-F. Error bar, mean ± standard error; *P* values are from *t* test (unpaired and two-tailed); **, $P \leq 0.005$; n, the number of quantified cells.

Figure S5

A

```
Human Tac (P01589)
MDSYLLMWGLLTFIMVPGCQAELCDDDPPEIPHATFKAMAYKEGTMLNCE
CKRGFRRIKSGSLYMLCTGNSSHSSWDNQCQCTSSATRNTTKQVTPQPEE
QKERKTTEMQSPMQPVDQASLPGHCREPPPWENEATERIYHFVVGQMVYY
QCVQGYRALHRGPAESVCKMTHGKTRWTQPQLICTGEMETSQFPGEEKPQ
ASPEGRPESETSCLVTTTDFQIQTEMAATMETSIFTTEYQVAVAGCVFLL
ISVLLLSGLTWQRRQRKSRRTI

GFP-Cstem-Tac(5A)
ss-GFP-
PAKPTTTPAPRPPTPAPTIASQPLSLRPEACRPAAGGAVHTRGLELCDDD
PPEIPHATFKAMAYKEGTMLNCECKRGFRRIKSGSLYMLCTGNSSHSSWD
NQCQCTSSATRNTTKQVTPQPEEQKERKTTEMQSPMQPVDQASLPGHCRE
PPPWENEATERIYHFVVGQMVYYQCVQGYRALHRGPAESVCKMTHGKTRW
TQPQLICTGEMETSQFPGEEKPQASPEGRPESETSCLVTTADFQIQAEMA
AAMETSIFAAEYQVAVAGCVFLLISVLLLSGLTWQRRQRKSRRTI

GFP-Cstem(7A)-Tac(5A)
ss-GFP-
PAKPAAAPAPRPPAPAPAIAAQPLALRPEACRPAAGGAVHTRGLELCDDD
PPEIPHATFKAMAYKEGTMLNCECKRGFRRIKSGSLYMLCTGNSSHSSWD
NQCQCTSSATRNTTKQVTPQPEEQKERKTTEMQSPMQPVDQASLPGHCRE
PPPWENEATERIYHFVVGQMVYYQCVQGYRALHRGPAESVCKMTHGKTRW
TQPQLICTGEMETSQFPGEEKPQASPEGRPESETSCLVTTADFQIQAEMA
AAMETSIFAAEYQVAVAGCVFLLISVLLLSGLTWQRRQRKSRRTI
```

B

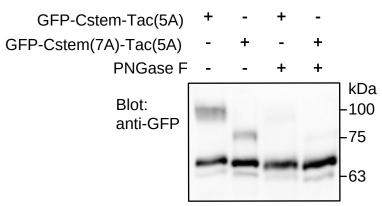

C

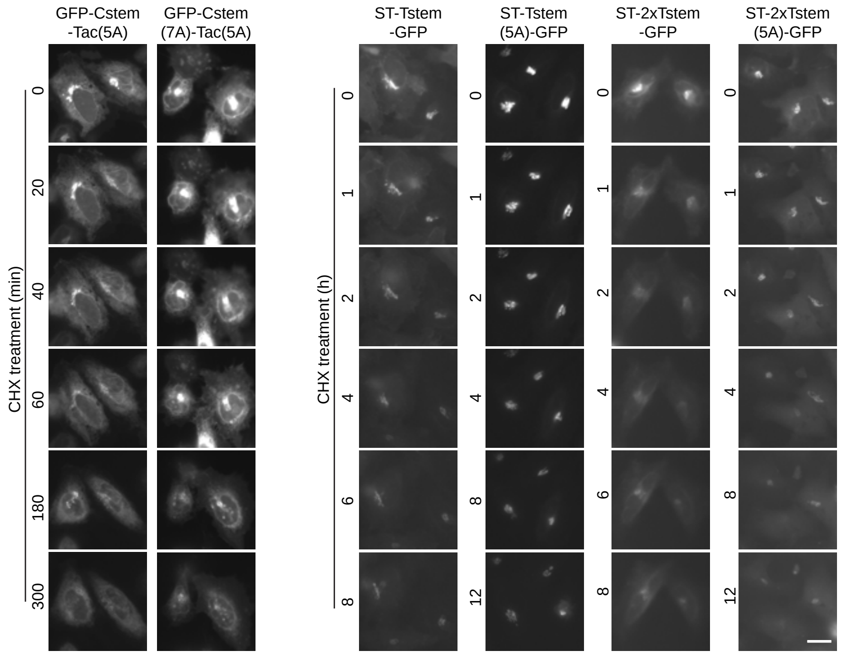

D

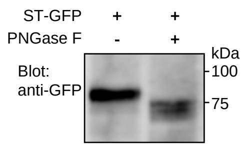

Figure S5

E

```
Human ST (P15907)
MIHTNLKKKFSCCVLVFLLFAVICVWKEKKKGSYYDSFKLQTKEFQVLKS
LGKLAMGSDSQSVSSSSTQDPHRGRQTLGSLRGLAKAKPEASFQVWNKDS
SSKNLIPRLQKIWKNYLSMNKYKVSYKGPGPGIKFSAEALRCHLRDHVNV
SMVEVTDFPFNTSEWEGYLPKESIRTKAGPWGRCAVVSSAGSLKSSQLGR
EIDDHDAVLRFNGAPTANFQQDVGTKTTIRLMNSQLVTTEKRFLKDSLYN
EGILIVWDPSVYHSDIPKWYQNPDYNFFNNYKTYRKLHPNQPFYILKPQM
PWELWDILQEISPEEIQPNPPSSGMLGIIIMMTLCDQVDIYEFLPSKRKT
DVCYYYQKFFDSACTMGAYHPLLYEKNLVKHLNQGTDEDIYLLGKATLPG
FRTIHC

ST-Tstem-moxGFP
MIHTNLKKKFSCCVLVFLLFAVICVWKEKKKGSYYDSFKLQTKEFQVLKS
LGKLAMGSDSPRLVTTTDFQIQTEMAATMETSIFTTEYQGSQSVSSSSTQ
DPHRGRQTLGSLRGLAKAKPEASFQVWNKDSSSKNLIPRLQKIWKNYLSM
NKYKVSYKGPGPGIKFSAEALRCHLRDHVNVSMVEVTDFPFNTSEWEGYL
PKESIRTKAGPWGRCAVVSSAGSLKSSQLGREIDDHDAVLRFNGAPTANF
QQDVGTKTTIRLMNSQLVTTEKRFLKDSLYNEGILIVWDPSVYHSDIPKW
YQNPDYNFFNNYKTYRKLHPNQPFYILKPQMPWELWDILQEISPEEIQPN
PPSSGMLGIIIMMTLCDQVDIYEFLPSKRKTDVCYYYQKFFDSACTMGAY
HPLLYEKNLVKHLNQGTDEDIYLLGKATLPGFRTIHC-(GFP)

ST-Tstem(5A)-moxGFP
MIHTNLKKKFSCCVLVFLLFAVICVWKEKKKGSYYDSFKLQTKEFQVLKS
LGKLAMGSDSPRLVTTADFQIQAEMAAAMETSIFAAEYQGSQSVSSSSTQ
DPHRGRQTLGSLRGLAKAKPEASFQVWNKDSSSKNLIPRLQKIWKNYLSM
NKYKVSYKGPGPGIKFSAEALRCHLRDHVNVSMVEVTDFPFNTSEWEGYL
PKESIRTKAGPWGRCAVVSSAGSLKSSQLGREIDDHDAVLRFNGAPTANF
QQDVGTKTTIRLMNSQLVTTEKRFLKDSLYNEGILIVWDPSVYHSDIPKW
YQNPDYNFFNNYKTYRKLHPNQPFYILKPQMPWELWDILQEISPEEIQPN
PPSSGMLGIIIMMTLCDQVDIYEFLPSKRKTDVCYYYQKFFDSACTMGAY
HPLLYEKNLVKHLNQGTDEDIYLLGKATLPGFRTIHC-(GFP)

ST-2xTstem-moxGFP
MIHTNLKKKFSCCVLVFLLFAVICVWKEKKKGSYYDSFKLQTKEFQVLKS
LGKLAMGSDSPRLVTTTDFQIQTEMAATMETSIFTTEYQGSQSVSSSSTQ
DPHRGRQTLGSLRGLAKAKPEASFQVWNKDSSSKNLIPRLQKIWKNYLSM
NKYKVSYKGPGPGIKFSAEALRCHLRDHVNVSMVEVTDFPFNTSEWEGYL
PKESIRTKAGPWGRCAVVSSAGSLKSSQLGREIDDHDAVLRFNGAPTANF
QQDVGTKTTIRLMNSQLVTTEKRFLKDSLYNEGILIVWDPSVYHSDIPKW
YQNPDYNFFNNYKTYRKLHPNQPFYILKPQMPWELWDILQEISPEEIQPN
PPSSGMLGIIIMMTLCDQVDIYEFLPSKRKTDVCYYYQKFFDSACTMGAY
HPLLYEKNLVKHLNQGTDEDIYLLGKATLPGFRTIHCAPVLVTTTDFQIQ
TEMAATMETSIFTTEYQ-(GFP)

ST-2xTstem(5A)-moxGFP
MIHTNLKKKFSCCVLVFLLFAVICVWKEKKKGSYYDSFKLQTKEFQVLKS
LGKLAMGSDSPRLVTTTDFQIQTEMAATMETSIFTTEYQGSQSVSSSSTQ
DPHRGRQTLGSLRGLAKAKPEASFQVWNKDSSSKNLIPRLQKIWKNYLSM
NKYKVSYKGPGPGIKFSAEALRCHLRDHVNVSMVEVTDFPFNTSEWEGYL
PKESIRTKAGPWGRCAVVSSAGSLKSSQLGREIDDHDAVLRFNGAPTANF
QQDVGTKTTIRLMNSQLVTTEKRFLKDSLYNEGILIVWDPSVYHSDIPKW
YQNPDYNFFNNYKTYRKLHPNQPFYILKPQMPWELWDILQEISPEEIQPN
PPSSGMLGIIIMMTLCDQVDIYEFLPSKRKTDVCYYYQKFFDSACTMGAY
HPLLYEKNLVKHLNQGTDEDIYLLGKATLPGFRTIHCAPVLVTTADFQIQ
AEMAAAMETSIFAAEYQ-(GFP)
```

F

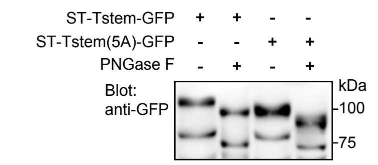

G

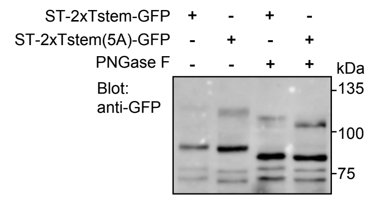

Figure S5

The sequences, gel migration profiles, and time-lapse images of Tac and ST chimeras. HeLa cells were used. (A) The sequences of Tac (Uniprot identifier: P01589) chimeras. Purple AAs, signal sequence (hereafter ss); blue AAs, cytosolic tail; grey shaded AAs, transmembrane domain; brown AAs, IBD; green shaded Ns, N-glycosylation sites; cyan shaded AAs, Tstem; underlined AAs, Cstem. In Tstem and Cstem, potentially O-glycosylated AAs (Thr or Ser) are shaded yellow and the corresponding Ala mutations are colored red. In chimera sequences, GFP is inserted between the ss and Cstem. (B) The gel migration profile of Tac chimeras demonstrates that Cstem likely undergoes the O-glycosylation. Lysates of cells transiently expressing indicated constructs were treated or not with PNGase F before immuno-blotting for GFP-tag. (C) The time-lapse images of Tac and ST chimeras. Cells transiently co-expressing GalT-mCherry and indicated construct were live-imaged in the presence of CHX. Scale bar, 20 µm. (D) The gel migration profile of ST demonstrates that it is likely N-glycosylated. The experimental procedure was similar to that of (B). (E) The sequences of ST (Uniprot identifier: P15907) chimeras. AAs are colored or shaded as described in (A) except that brown AAs indicate the catalytic domain. GFP is appended at the C-terminus of chimeras. (F,G) The gel migration profiles demonstrate that Tstem is likely glycosylated as designed in ST-Tstem-GFP and ST-2xTstem-GFP. The experiments were conducted as described in (B). In (B,D,F,G), molecular weight (kDa) is indicated at the right side.

Figure S6

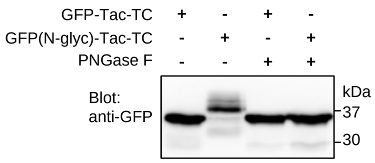

Figure S6

GFP(N-glyc)-Tac-TC is N-glycosylated. HeLa cells transiently expressing indicated construct were lysed and treated or not with PNGase F before gel separation and immuno-blotting for GFP-tag. Molecular weight (kDa) is indicated at the right side.

Figure S7

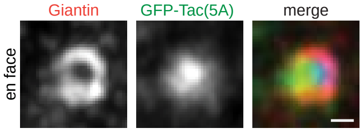

Figure S7

GFP-Tac(5A) localizes to the interior of the *trans*-Golgi cisternae. The experiment is similar to that of Figure 7D. Scale bar, 500 nm.

Figure S8

Fig. S3A

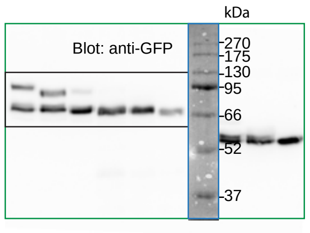

Fig. S3B

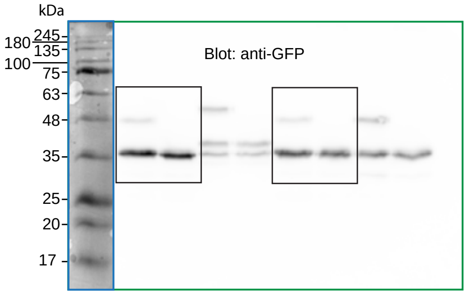

Fig. S5B

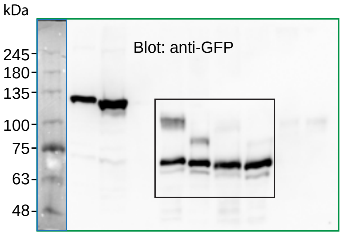

Fig. S5D

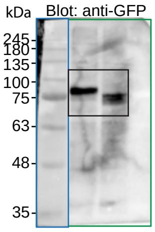

Fig. S5F

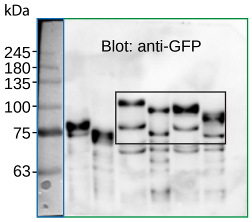

Fig. S5G

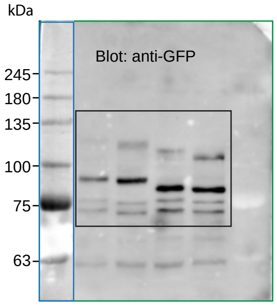

Fig. S6

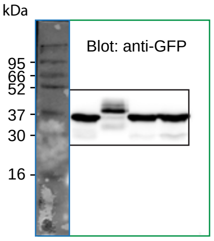

Figure S8

Uncropped gel images. Green box, the chemiluminescence image of the whole gel blot; black box, the cropped region for figure preparation; blue box, white light image of molecular weight marker bands, which are used to label the molecular weight (kDa).

Supplementary Table 1
List of DNA plasmids

name	*vector (source)*	*cloning sites*	*primers*	*brief method or source reference*
Tac-GFP-intermediate	pEGFP-N1 (Clontech)	XhoI/EcoRI		It was previously described (44).
GFP-Tac	pEGFP-C1 (Clontech)	NheI/EcoRI	Primer pair #1: 5'- AGT GAC GCT AGC GCC ACC ATG GAT TCA TAC CTG CTG ATG TGG -3' and 5'- CAC CCC GGT GAA CAG CTC CTC GCC CTT GCT CAC CAT ACC GGT TGC CTG GCA GCC AGG CAC -3'. Primer pair #2: 5'- GTG CCT GGC TGC CAG GCA ACC GGT ATG GTG AGC AAG GGC GAG GAG -3' and 5'- CGG GTC ATC GTC ACA GAG CTC CTC GAG CTT GTA CAG CTC GTC CAT GCC -3'. Primer pair #3: 5'- GGC ATG GAC GAG CTG TAC AAG CTC GAG GAG CTC TGT GAC GAT GAC CCG -3' and 5'- AGT GAC GAA TTC CTA GAT TGT TCT TCT ACT CTT CCT C -3'.	Fragment 1 consisting of the signal peptide was PCR amplified from Tac-GFP-intermediate using the primer pair #1. Fragment 2 consisting of the CDS of GFP was PCR amplified from pEGFP-N1 vector using the primer pair #2. Fragment 3 consisting of the CDS of Tac was PCR amplified from Tac-GFP-inermediate. Finanlly, the construct was assembled by the overlapping PCR of three fragments using the first and last listed primers and the PCR product was digested by NheI/EcoRI and ligated into pEGFP-C1 vector using the same sites.
GFP-Tac-STC	GFP-Tac	XhoI/EcoRI	5'- AGT GAC CTC GAG CTC GTC ACA ACA ACA GAT TTT C -3' and 5'- AGT GAC GAA TTC CTA GAT TGT TCT TCT ACT CTT CCT C -3'	The CDS was PCR amplified from GFP-Tac using the listed primer pair. The PCR product was digested by XhoI/EcoRI and ligated into GFP-Tac using the same sites.
GFP-Tac-STC(5A)	GFP-Tac	XhoI/EcoRI	5'- AGT GAC CTC GAG CTC GTC ACA ACA GCA GAT TTT C -3' and 5'- AGT GAC GAA TTC CTA GAT TGT TCT TCT ACT CTT CCT C -3'	The CDS was PCR amplified from GFP-Tac(5A) using the listed primer pair. The PCR product was digested by XhoI/EcoRI and ligated into GFP-Tac using the same sites.
GFP-Tac-TC	GFP-Tac	XhoI/EcoRI	5'- AGT GAC CTC GAG GTA GCA GTG GCC GGC TGT GTT TTC CTG -3' and 5'- AGT GAC GAA TTC CTA GAT TGT TCT TCT ACT CTT CC -3'	The CDS was PCR amplified from GFP-Tac using the listed primer pair. The PCR product was digested by XhoI/EcoRI and ligated into GFP-Tac using the same sites.
GFP-Tac(5A)	GFP-Tac	XhoI/EcoRI	Primer pair #1: 5'- GGC ATG GAC GAG CTG TAC AAG CTC GAG GAG CTC TGT GAC GAT GAC CCG -3' and 5'- CAT GGC TGC AGC CAT TTC TGC CTG TAT TTG AAA ATC TGC TGT TGT GAC GAG GCA GGA AG -3'; Prime pair #2: 5'- GCA GAA ATG GCT GCA GCC ATG GAG ACG TCC ATA TTT GCA GCA GAG TAC CAG GTA GCA GTG -3' and 5'- AGT GAC GAA TTC CTA GAT TGT TCT TCT ACT CTT CCT C -3'.	Two PCRs were conducted using GFP-Tac as the template and Primer pair #1 and #2. The resulting two PCR fragments were mixed and subjected to the final PCR amplification using the first and last listed primers. The final PCR product was digested by XhoI/EcoRI and ligated into GFP-Tac using the same sites.
Tac-GFP	GFP-Tac	AgeI/EcoRI	Primer pair #1: 5'-AGT GAC ACC GGT GAG CTC TGT GAC GAT GAC CCG-3' and 5'- CTC GCC CTT GCT CAC CAT CTC GAG GAT TGT TCT TCT ACT CTT CC -3'; Primer pair #2: 5'- GG AAG AGT AGA AGA ACA ATC CTC GAG ATG GTG AGC AAG GGC GAG -3' and 5'-AGT GAC GAA TTC CTA CTT GTA CAG CTC GTC CAT GCC -3'.	Two PCRs were conducted using GFP-Tac as the template and Primer pair #1 and #2. The resulting two PCR fragments were mixed and subjected to the final PCR amplification using the first and last listed primers. The final PCR product was digested by AgeI/EcoRI and ligated into GFP-Tac using the same sites.
Tac(5A)-GFP	Tac-GFP	AgeI/XhoI	5'-AGT GAC ACC GGT GAG CTC TGT GAC GAT GAC CCG-3' and 5'- CTC GCC CTT GCT CAC CAT CTC GAG GAT TGT TCT TCT ACT CTT CC -3'	The CDS comprising Tac(5A) was PCR amplified from GFP-Tac(5A) using the listest primer pair. The PCR product was digested by AgeI/XhoI and ligated into Tac-GFP using the same sites.
GFP(*N*-glyc)-Tac-TC	GFP-Tac	AgeI/XhoI	Primer pair #1: 5'- GTG CCT GGC TGC CAG GCA ACC GGT ATG GTG AGC AAG GGC GAG GAG -3', 5'- CTC CAG CTT GTG CCC CAG GAT GCT GCC GTT CTC CTT GAA GTC GAT GCC C -3' and Primer pair #2:5'- G GGC ATC GAC TTC AAG GAG AAC GGC AGC ATC CTG GGG CAC AAG CTG GAG -3', 5'- CGG GTC ATC GTC ACA GAG CTC CTC GAG CTT GTA CAG CTC GTC CAT GCC-3'	Two PCRs were conducted using GFP-Tac as the template and Primer pair #1 and #2. The resulting two PCR fragments were mixed and subjected to the final PCR amplification using the first and last listed primers. The final PCR product was digested by AgeI/XhoI and ligated into GFP-Tac using the same sites.
GFP-Cstem-Tac(5A)	GFP-Tac	XhoI/EcoRI	Primer pair #1: 5'- AGT GAC CTC GAG CCA GCG AAG CCC ACC ACG ACG -3', 5'- CGG GTC ATC GTC ACA GAG CTC CAG CCC CCT CGT GTG CAC -3' and Primer pair #2: 5'- GTG CAC ACG AGG GGG CTG GAG CTC TGT GAC GAT GAC CCG -3', 5'- AGT GAC GAA TTC CTA GAT TGT TCT TCT ACT CTT CCT C -3'	Two PCRs were conducted using CD8a and GFP-Tac(5A) as the template and Primer pair #1 and #2 respectively. The resulting two PCR fragments were mixed and subjected to the final PCR amplification using the first and last listed primers. The final PCR product was digested by XhoI/EcoRI and ligated into GFP-Tac using the same sites.
GFP-CD8a(7A)	GFP-Tac	XhoI/EcoR	Primer pair #1: 5'-AGT GAC CTC GAG AGC CAG TTC CGG GTG TCG CCG-3' and 5'- GGG CGC CGG TGC TGG TGG TCG CGG CGC TGG AGC TGC TGC GGG CTT CGC TGG CAG GAA GAC -3'; Primer pair #2: 5'- CCA CCA GCA CCG GCG CCC GCA ATC GCG GCA CAG CCC CTG GCA CTG CGC CCA GAG GCG TGT -3', 5'- AGT GAC GAA TTC TTA GAC GTA TCT CGC CGA AAG GCT GGG -3'.	Two PCRs were conducted using CD8a as the template and Primer pair #1 and #2. The resulting two PCR fragments were mixed and subjected to the final PCR amplification using the first and last listed primers. The final PCR product was digested by XhoI/EcoRI and ligated into GFP-Tac using the same sites.

GFP-Cstem(7A)-Tac(5A)	GFP-Tac	XhoI/EcoR	Primer pair #1: 5'-AGT GAC CTC GAG CCA GCG AAG CCC GCA GCA GC-3' and 5'- CGG GTC ATC GTC ACA GAG CTC CAG CCC CCT CGT GTG CAC -3'; Primer pair #2: 5'- GTG CAC ACG AGG GGG CTG GAG CTC TGT GAC GAT GAC CCG -3' and 5'- AGT GAC GAA TTC CTA GAT TGT TCT TCT ACT CTT CCT C -3'.	Two PCRs were conducted using GFP-CD8a(7A) and GFP-Tac(5A) as the template and Primer pair #1 and #2 respectively. The resulting two PCR fragments were mixed and subjected to the final PCR amplification using the first and last listed primers. The final PCR product was digested by XhoI/EcoRI and ligated into GFP-Tac using the same sites.
ST-GFP	pmoxGFP-N1	XhoI/BamH I	5'-AGT GAC CTC GAG GCC ACC ATG ATT CAC ACC AAC CTG-3' and 5'-AGT GAC GGA TCC CGG CAG TGA ATG GTC CGG AAG-3'	The CDS of ST6GAL1 was PCR amplified from an IMAGE clone (GenBank Accession No.: BC040009). The PCR product was digested by XhoI/BamHI and ligated into pmoxGFP-N1 (Addgene plasmid # 68070) using the same sites.
ST-Tstem-GFP	ST-GFP	XhoI/AgeI	Primer pair #1: 5'- AGT GAC CTC GAG GCC ACC ATG ATT CAC ACC AAC CTG AAG -3' and 5'- ATC TGT TGT TGT GAC GAG CCG CGG GGA ATC AGA CCC CAT GGC -3'; Primer pair #2: 5'- GCC ATG GGG TCT GAT TCC CCG CGG CTC GTC ACA ACA ACA GAT -3' and 5'- GCT TGA GGA TAC AGA CTG GGA TCC CTG GTA CTC TGT TGT AAA TAT GG -3'; Primer pair #3: 5'- CC ATA TTT ACA ACA GAG TAC CAG GGA TCC CAG TCT GTA TCC TCA AGC -3' and 5'- AG TGA CAC CGG TGC GCA GTG AAT GGT CCG GAA GCC -3'.	Fragment 1 consisting of the cytosolic tail, transmembrane domain, and stem region of ST was PCR amplified from ST-GFP using Primer pair #1. Fragment 2 consisting of the stem region of Tac was PCR amplified from GFP-Tac using Primer pair #2. Fragment 3 consisting of the remaing fragment of ST was PCR amplified from ST-GFP using Primer pair #3. Finanlly, the overlapping PCR of three fragments using the first and last listed primers was conducted and the PCR product was digested by XhoI/AgeI and ligated into ST-GFP using the same sites.
ST-Tstem(5A)-GFP	ST-GFP	XhoI/AgeI	Primer pair #1: 5'- AGT GAC CTC GAG GCC ACC ATG ATT CAC ACC AAC CTG AAG -3' and 5'- G AAA ATC TGC TGT TGT GAC GAG CCG CGG GGA ATC AGA CCC CAT GGC -3'; Primer pair #2: 5'- GCC ATG GGG TCT GAT TCC CCG CGG CTC GTC ACA ACA GCA GAT TTT C -3' an d 5'- C ATA TTT GCA GCA GAG TAC CAG GGA TCC CTG GTA CTC TGT TGT AAA TAT GG -3'; Primer pair #3: 5'- CC ATA TTT ACA ACA GAG TAC CAG GGA TCC CTG GTA CTC TGC TGC AAA TAT G -3' and 5'- AG TGA CAC CGG TGC GCA GTG AAT GGT CCG GAA GCC -3'.	Fragment 1 consisting of the cytosolic tail, transmembrane domain, and stem region of ST was PCR amplified from ST-GFP using Primer pair #1. Fragment 2 consisting of the stem region of Tac was PCR amplified from GFP-Tac(5A) using Primer pair #2. Fragment 3 consisting of the remaing fragment of ST was PCR amplified using ST-GFP as the template and the primer pair #3. Finanlly, the overlapping PCR of three fragments using the first and last listed primers was conducted and the PCR product was digested by XhoI/AgeI and ligated into ST-GFP using the same sites.
ST-2xTstem-GFP	ST-Tstem-GFP	AgeI/NotI	Primer pair #1: 5'- A GTG ACA CCG GTC CTC GTC ACA ACA ACA GAT TTT C -3', 5'- CTC GCC CTT GGA CAC CAT GGT GGC CTG GTA CTC TGT TGT AAA TAT GG -3' and primer pair #2: 5'- CC ATA TTT ACA ACA GAG TAC CAG GCC ACC ATG GTG TCC AAG GGC GAG -3', 5'- AGT GAC GCG GCC GCT TTA CTT GTA CAG CTC GTC CAT -3'.	Two PCRs were conducted using GFP-Tac and ST-GFP as the template and Primer pair #1 and #2 respectively. The resulting two PCR fragments were mixed and subjected to the final PCR amplification using the first and last listed primers. The final PCR product was digested by AgeI/NotI and ligated into ST-Tstem-GFP using the same sites.
ST-2xTstem(5A)-GFP	ST-Tstem-GFP	AgeI/NotI	Primer pair #1: 5'- A GTG ACA CCG GTC CTC GTC ACA ACA GCA GAT TTT C -3', 5'- CTC GCC CTT GGA CAC CAT GGT GGC CTG GTA CTC TGC TGC AAA TAT GG -3' and primer pair #2: 5'- CC ATA TTT GCA GCA GAG TAC CAG GCC ACC ATG GTG TCC AAG GGC GAG -3', 5'- AGT GAC GCG GCC GCT TTA CTT GTA CAG CTC GTC CAT -3'.	Two PCRs were conducted using GFP-Tac(5A) and ST-GFP as the template and Primer pair #1 and #2 respectively. The resulting two PCR fragments were mixed and subjected to the final PCR amplification using the first and last listed primers. The final PCR product was digested by AgeI/NotI and ligated into ST-Tstem-GFP using the same sites.
SBP-GFP-Tac	SBP-GFP-E-cadherin	FseI/XhoI	5'- AGT GAC GGC CGG CCA GAC GAG CTC TGT GAC GAT GAC CCG CC -3' and 5'- AGT GAC CTC GAG CTA GAT TGT TCT TCT ACT CTT CCT C -3'	The CDS comprising mature Tac sequence was PCR amplified from GFP-Tac using the listed primer. The PCR product was digested by FseI/XhoI and ligated into SBP-GFP-E-cadherin using the same sites.
SBP-GFP-Tac-TC	SBP-GFP-E-cadherin	FseI/XhoI	5'- AGT GAC GGC CGG CCA GAC GTA GCA GTG GCC GGC TGT GTT TTC -3' and 5'- AGT GAC CTC GAG CTA GAT TGT TCT TCT ACT CTT CCT C -3'	The CDS comprising Tac-TC was PCR amplified from GFP-Tac using the listed primer. The PCR product was digested by FseI/XhoI and ligated into SBP-GFP-E-cadherin using the same sites.
His-mCherry	pET-30ax	EcoRI/Bam HI	5'- AGT GAC GAA TTC ATG GTG AGC AAG GGC GAG GAG G -3' and 5'- AGT GAC GGA TCC TTA CTT GTA CAG CTC GTC CAT GCC -3'	The CDS comprising mCherry was PCR amplified from GalT-mCherry using the listest primer pair. The PCR product was digested by EcoRI/BamHI and ligated into pET30ax using the same sites.
GST-mCherry	pGEB	EcoRI/Bam HI		The insert-mCherry was released from EcoRI/BamHI digestted His-mCherry and ligated into pGEB using the same sites.
CD8a-GFP				It was previously described (44).
CD8a				It was previously described (45).
GalT-GFP				A gift from J. Lippincott-Schwartz.
GFP-GM130				A gift from M. De Matties (46).
GalT-mCherry				It was previously described (47).
Sec61β-mCherry				It was previously described (36).
CD8a-furin-mEos2				It was previously described (48).
pET-30ax				It was previously described (49).
SBP-mCherry-GPI				A gift from F. Perez (25).
TNFα-SBP-GFP				A gift from F. Perez (25).
VSVG-SBP-GFP				A gift from F. Perez (25).

SBP-GFP-E-cadherin				A gift from F. Perez (25).
GFP-ERGIC53				A gift from H. Hauri (50).
TfR-GFP				A gift from T. Kirchhausen.
pmoxGFP-N1				A gift from E. Snapp (Addgene plasmid # 68070)
